# Spatiotemporal Variation of Microbial Communities in the Ultra-Oligotrophic Eastern Mediterranean Sea

**DOI:** 10.3389/fmicb.2022.867694

**Published:** 2022-04-07

**Authors:** Markus Haber, Dalit Roth Rosenberg, Maya Lalzar, Ilia Burgsdorf, Kumar Saurav, Regina Lionheart, Yoav Lehahn, Dikla Aharonovich, Laura Gómez-Consarnau, Daniel Sher, Michael D. Krom, Laura Steindler

**Affiliations:** ^1^Department of Marine Biology, Leon H. Charney School of Marine Sciences, University of Haifa, Haifa, Israel; ^2^Department of Aquatic Microbial Ecology, Institute of Hydrobiology, Biology Centre CAS, České Budějovice, Czechia; ^3^Bioinformatics Service Unit, University of Haifa, Haifa, Israel; ^4^The Dr. Moses Strauss Department of Marine Geosciences, Leon H. Charney School of Marine Sciences, University of Haifa, Haifa, Israel; ^5^Department of Biological Sciences, University of Southern California, Los Angeles, CA, United States; ^6^Department of Biological Oceanography, Centro de Investigación Científica y de Educación Superior de Ensenada, Ensenada, Mexico; ^7^Morris Kahn Marine Research Station, Environmental Geochemistry Lab., Leon H. Charney School of Marine Sciences, University of Haifa, Haifa, Israel

**Keywords:** Mediterranean Sea, SAR11, transect, seasonality, 16S rRNA

## Abstract

Marine microbial communities vary seasonally and spatially, but these two factors are rarely addressed together. In this study, the temporal and spatial patterns of the bacterial and archaeal community were studied along a coast-to-offshore transect in the Eastern Mediterranean Sea (EMS) over six cruises, in three seasons of 2 consecutive years. Amplicon sequencing of 16S rRNA genes and transcripts was performed to determine presence and activity, respectively. The ultra-oligotrophic status of the Southeastern Mediterranean Sea was reflected in the microbial community composition dominated by oligotrophic bacterial groups such as SAR11, even at the most coastal station sampled, throughout the year. Seasons significantly affected the microbial communities, explaining more than half of the observed variability. However, the same few taxa dominated the community over the 2-year sampling period, varying only in their degree of dominance. While there was no overall effect of station location on the microbial community, the most coastal site (16 km offshore) differed significantly in community structure and activity from the three further offshore stations in early winter and summer. Our data on the microbial community compositions and their seasonality support previous notions that the EMS behaves like an oceanic gyre.

## Introduction

Marine microbial communities play a pivotal function in the ocean’s biogeochemistry because of their key roles in the carbon, nitrogen, and sulfur cycles (Falkowski et al., 2008). Their composition is strongly affected by seasons with recurring microbial turnover over different years, as revealed by oceanographic time-series studies conducted both at offshore stations [e.g., Bermuda Atlantic Time-Series Study (BATS) in the Western Atlantic Ocean and the Hawaii Ocean Time-series (HOTS) in the North Pacific subtropical gyre; Giovannoni and Vergin, 2012] and more coastal affected sites [e.g., the San Pedro Ocean Time Series (SPOTS) in the Southern California Bight: Fuhrman et al., 2006; the western English Channel: Gilbert et al., 2012; and the Northwestern Mediterranean Sea: Galand et al., 2018; Auladell et al., 2021]. In addition, spatial variability of marine surface water microbial communities has been reported at various scales, ranging from a distance of a few kilometers between sampling sites to samples collected in different ocean basins (Ghiglione et al., 2005; Sunagawa et al., 2015; Sebastián et al., 2021). The observed differences in these studies were linked to gradients in environmental conditions (Fortunato et al., 2011; Sunagawa et al., 2015; Wang et al., 2019) and the presence of distinct water masses with different physicochemical properties (Dubinsky et al., 2017; Morales et al., 2018). Spatial variability is especially evident when comparing nearshore and offshore microbial communities due to gradients including nutrient availability, temperature, dissolved organic matter, and, especially within estuaries, salinity change from coastal to the open ocean waters (Ghiglione et al., 2005; Fortunato et al., 2011; Quero and Luna, 2014; Lucas et al., 2016; Wang et al., 2019). In addition to these parameters, factors such as light and rainfall are also influenced by season leading to weakening or strengthening environmental gradients along coast-to-offshore transects. To date, few studies have focused on the understanding of the dynamics of microbial communities both in seasons and along environmental gradients from coastal to offshore waters (Fortunato et al., 2011; Lucas et al., 2016; Wang et al., 2019). Both Lucas et al. (2016) and Fortunato et al. (2011) analyzed microbial communities along an estuary to open-sea transect. Lucas et al. (2016) observed a clear separation between the microbial communities of open water and estuarine influenced sites. Temperature had the strongest influence on the microbial community composition; however, data analysis was limited to presence/absence of bacterial groups due to the use of automated ribosomal intergenic spacer analysis (ARISA). Fortunato et al. (2011) found that abiotic factors, especially salinity, structured the microbial community along the transect from the Colombia river to offshore into five groups. When analyzing each spatial group separately, they detected seasonal influences on the microbial community composition. Only Wang et al. (2019) investigated changes in the community structure in the absence of a strong salinity gradient. Still, they found a stable separation in nearshore, continental shelf, and offshore groups along the transect from the Piver’s Island Coastal Observatory (PICO) time-series site to the oligotrophic waters of the Sargasso Sea. Distance from shore and temperature had the largest effect on the separation of the microbial communities into the three observed groups.

Here, we investigated the seasonal dynamics of the bacterial and archaeal microbial communities along a coast-to-offshore transect in the ultra-oligotrophic Eastern Mediterranean Sea (EMS). This mostly land-enclosed region represents one of the largest water bodies severely depleted in phosphate (Krom et al., 1991) and one of the most oligotrophic oceanic regions on Earth (Krom et al., 2010). Despite being an inland sea, it has been suggested to behave like an open ocean gyre (Powley et al., 2017). The nutrient cycle at offshore sites in the EMS exhibits predictable patterns. The water column is well stratified during summer with a distinct deep chlorophyll maximum (DCM) and very low nutrient content in the surface waters (Kress and Herut, 2001; Krom et al., 2014; Ben Ezra et al., 2021). In winter, because of increasingly deep-water mixing, nutrient-enriched water is advected into the photic zone. Due to local weather conditions in this region (short cold and wet, often stormy, periods interspersed with clear sunny ones), the phytoplankton bloom starts soon after the nutrients are supplied to the photic zone and develops throughout the winter, reaching maximum chlorophyll levels in late winter (Krom et al., 2014). Most of the dissolved phosphate in surface waters is consumed during the winter phytoplankton bloom, while measurable nitrate persists (Ben Ezra et al., 2021). In the summer, autotrophs in offshore waters of this region tend to be phosphate and nitrogen co-limited, while heterotrophic bacteria are either phosphate or phosphate and nitrogen co-limited (Thingstad et al., 2005; Tanaka et al., 2011; Tsiola et al., 2016). Thus, the marked seasonality of the region is likely to shape the pattern of microbial communities significantly. However, detailed molecular characterizations of these communities are limited to a few studies that represent only snapshots of the communities taken at a single time point (Feingersch et al., 2010; Keuter et al., 2015; Dubinsky et al., 2017; Sebastián et al., 2021), with one exception that investigated the microbial community composition through time and across different depths at an offshore site (Roth Rosenberg et al., 2021).

The main goal of the present study was to examine the effects of distance from shore across a transect together with seasonality on both microbial composition and activity in the EMS. We collected surface seawater (10 m) in six cruises along a coastal-to-offshore transect in three seasons (early winter, spring, and summer) over 2 consecutive years (2014–2016). The microbial community structure and potential of activity were determined by amplicon sequencing of 16S rRNA genes and transcripts (16S rRNA amplification on cDNA templates), respectively, following previous studies (e.g., Campbell et al., 2011; Campbell and Kirchman, 2013). Environmental influences on the bacterial and archaeal community were then determined from the measured physical, chemical, and remote sensing parameters. The phytoplankton community structure was characterized based on flow cytometry and pigment analysis.

## Materials and Methods

### Sampling

**Figure 1:**
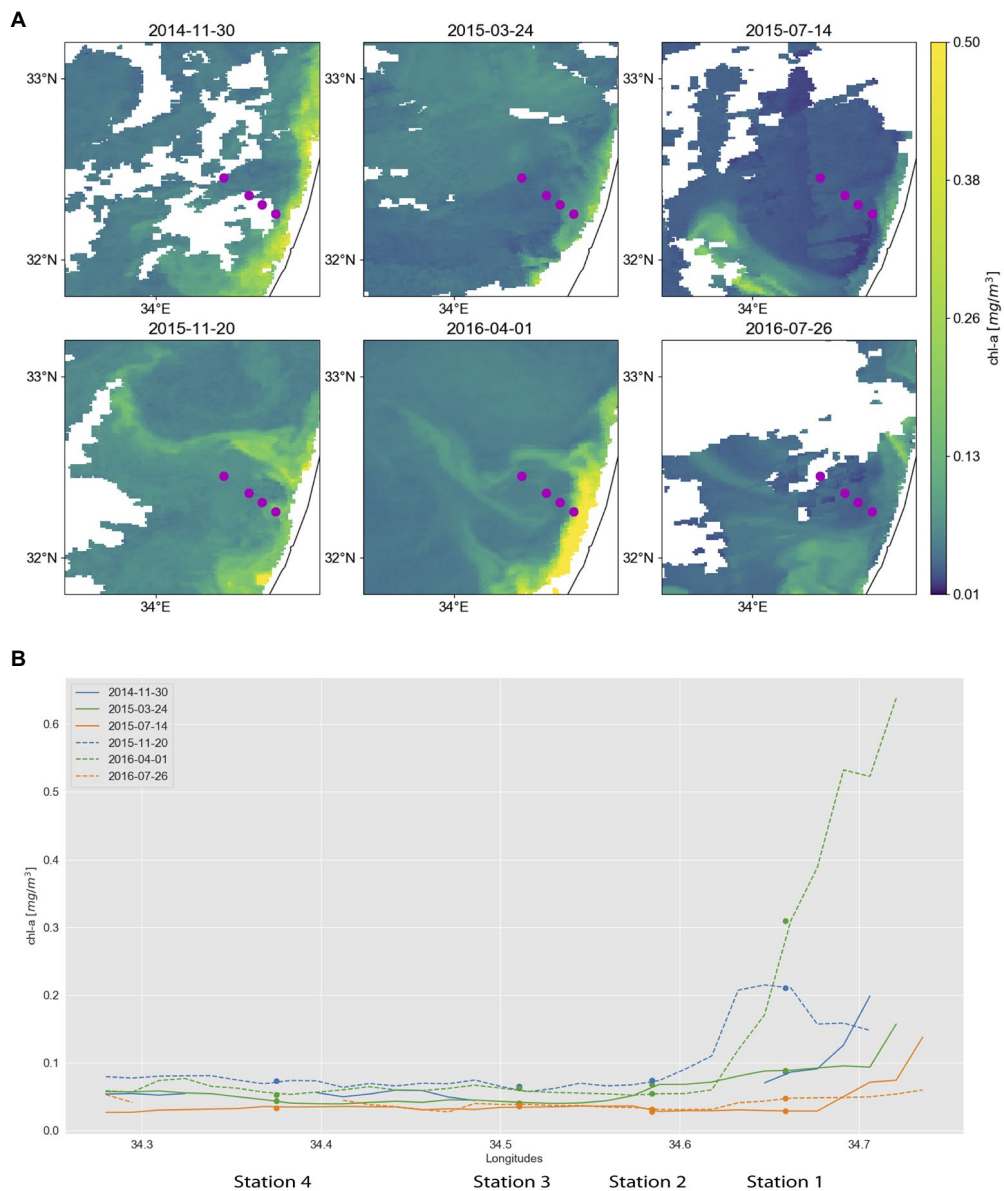
Surface chlorophyll concentrations in the southeastern Mediterranean. Dots mark locations of sampling stations, which are from right (coast) to left (offshore): Station 1, 2, 3, and 4. **(A)** Maps derived from Copernicus Marine Environment Monitoring Service (CMEMS) merged satellite data. Purple dots mark the locations of sampling stations. **(B)** Surface chlorophyll concentrations along a coast-to-open-sea transect overlapping the sampling stations. The transects are derived from the multi-satellite chlorophyll maps shown in A, with each line corresponding to a different sampling date. To reduce the area masked by clouds, chlorophyll maps from 30.11.2014, 20.11.2015, 01.04.2016, and 26.07.2016 were used instead of 01.12.2014, 17.11.2015, 30.03.2016, and 25.07.2016, respectively. Furthermore, maps for 30.11.2014, 24.3.2015, 14.7.2015, and 26.7.2016 are composed of 5, 3, 3, and 3 consecutive images, respectively.

Six one-day cruises were performed onboard the R/V Mediterranean Explorer over a 2-year period: two in early winter (December 1st, 2014; November 17th, 2015), two in spring (March 24th, 2015; March 30th, 2016), and two in summer (July 14th, 2015; July 25th, 2016). For each cruise, four stations were sampled (labeled in ascending order with distance from shore) along a coastal-to-offshore transect from the Herzliya marina, Israel (Figure 1). At each station, we measured the vertical profile of *in situ* parameters up to a depth of 500 m using a conductivity, temperature, and depth (CTD) probe (SeaBird CTD profiler SBE 19plus V2), a fluorescence probe (Seapoint fluorometer) to measure chlorophyll *a* and turbidity with a Seapoint turbidity sensor. The fluorometer was calibrated with bottle chlorophyll measurements before each cruise. Both the fluorometer and the turbidity sensor were mounted on the CTD. Data were extracted using the Seasoft V2 software suite and plotted using Ocean Data View 4.7.4 (Schlitzer, 2015).^1^

Seawater for nutrient, phytoplankton pigments, cell counts, DNA, and RNA analysis was collected from 10 m depth using a 12-bottle rosette sampler equipped with 8 L Niskin bottles. To better identify gradients along the transect, CTD and nutrient data were collected for two additional stations: one located between stations 1 and 2, and the other between stations 2 and 3. Station coordinates, bottom depths, and distance between stations and the shore are summarized in Table 1.

**Table 1:** Overview of the occurrence and abundance of ESVs grouped into four categories: dominant: ≥1% relative abundance in 75% of the samples, rare: always <1% relative abundance, intermediate persistent: present in ≥75% of the samples, intermediate fluctuating <75% of the samples.

Group	# ESV	Average occurrence (%)	Average abundance ± SD (%)			Top 5	Top 5 > twice
			Winter	Spring	Summer		
DNA							
Dominant	8	100.0	35.2 ± 6.9	32.0 ± 3.5	48.4 ± 5.2	6	6
Intermediate persistent	37	93.8	24.5 ± 3.0	27.5 ± 1.3	26.1 ± 2.3	2	1
Intermediate fluctuating	25	43.2	7.6 ± 2.8	4.3 ± 0.9	4.8 ± 2.7	1	0
Rare	1,590	13.2	32.7 ± 7.2	36.2 ± 3.0	20.7 ± 3.2	0	0
RNA							
Dominant	5	100.0	19.0 ± 6.5	14.1 ± 3.5	23.8 ± 3.9	5	4
Intermediate persistent	34	94.0	23.2 ± 8.7	42.3 ± 4.6	34.2 ± 5.5	10	6
Intermediate fluctuating	51	37.1	18.8 ± 9.3	6.3 ± 2.2	15.2 ± 9.2	17	0
Rare	2,428	12.0	39.0 ± 7.9	37.3 ± 2.6	26.9 ± 1.5	0	0

Top5 describes how many of the ESVs of one group were at least once among the five most abundant ESVs of a sample. Top5 > twice described how many were among the top5 in more than two samples. The category for each ESV can be found in the ESV table (Supplementary File 1 sheet 1).

### Surface Chlorophyll a Maps

Satellite-based maps of surface chlorophyll concentration are derived from the Copernicus Marine Environment Monitoring Service (CMEMS).^2^ We used the level-3 Mediterranean Sea reprocessed surface chlorophyll concentration product, consisting of merged SeaWiFS, MODIS-Aqua, MERIS, and VIIRS satellite data (OCEANCOLOUR_MED_CHL_L3_REP_OBSERVATIONS_009_073). Using multi-satellite data allows continuous tracking of fine-scale chlorophyll filaments as they are advected and deformed by the currents (Lehahn et al., 2017). Surface chlorophyll concentrations are estimated *via* the MedOC4 (Volpe et al., 2007) and the AD4 (D’Alimonte and Zibordi, 2003) algorithms for case-1 and case-2 waters, respectively. Spatial and temporal resolution is 1 km and 1 day, respectively. To reduce the area masked by clouds, chlorophyll maps from 30.11.2014, 20.11.2015, 01.04.2016, to 26.07.2016 were used instead of 01.12.2014, 17.11.2015, 30.03.2016, and 25.07.2016, respectively. Furthermore, maps for 30.11.2014, 24.3.2015, 14.7.2015, and 26.7.2016 are composed of 5, 3, 3, and 3 consecutive images, respectively.

### Nutrient Analysis

Seawater for nutrient analysis was collected in 15 ml Falcon tubes pre-rinsed with sample seawater. For each nutrient, duplicate non-filtered samples were frozen onboard directly after collection and kept at −20°C until analysis. Silicate, nitrate + nitrite, and soluble reactive phosphate content were measured within 6 weeks calorimetrically on a LACHAT Instruments Quik-Chem 8,500 Flow Injection Analyzer (Grasshoff et al., 2009) at the service unit of the Interuniversity Institute for Marine Sciences in Eilat, Israel. The internal precision (the average deviation from the mean of duplicate samples) of the measurements and the effective limit of detection for these frozen samples were 0.04 μmol/L for phosphate and 0.05 μmol/L for both silicate and nitrate + nitrite, respectively. Accuracy was obtained by calibration against a suite of solutions prepared by dilution of commercial, high-concentration standards (1,000 mg/L, Merck). The calibration curve was run several times during every flow injection analyzer session.

During the July 2016 cruise, phosphate, nitrate, and in addition ammonia were also measured in non-frozen samples. These samples were filtered through 0.4 μm filters, transferred into 15 ml Falcon tubes, stored at 4°C, and analyzed within 24 h at the Morris Kahn Marine Laboratory, Sdot Yam, Israel, using a SEAL AA-3 autoanalyzer. Samples for silicate analysis from these samples were acidified and kept frozen until 5 days before analysis. Silicate, nitrate + nitrite, and soluble reactive phosphate were measured calorimetrically following the manufacturer’s operating protocols (SEAL version 2015). Silicate was measured by a modification of the silico-molybdate blue method (Grasshoff et al., 1983), nitrate + nitrite determined by a purple diazo dye after Cd reduction (Armstrong et al., 1967), while soluble reactive phosphate was determined using a long flow cell (50 cm) and a modified Murphy and Riley (1962) molybdophosphate blue method. Ammonia was determined using a fluorometric method based on Kérouel and Aminot (1997). The precision of these methods at the used range were 33, 21, 2, and 6 nM, respectively, and the limits of detection were 55, 63, 2, and 5 nM (defined as three times the precision of the seawater blank).

### Flow Cytometry

For flow cytometry sample collection, 1.5 ml triplicate seawater samples were fixed with glutaraldehyde (0.125% final concentration), incubated in the dark for 10 min, stored in liquid nitrogen onboard, and kept at −80°C in the lab until analysis. For analysis, samples were thawed at room temperature and run on a BD FACSCanto™ II Flow Cytometry Analyzer Systems (BD Biosciences) with 2 μm diameter fluorescent beads (Polysciences, Warminster, PA, United States) added as an internal standard. Samples were run first without staining to use the natural fluorescence of the cells (chlorophyll and phycoerythrin pigments) for identification of three cell types: (i) *Prochlorococcus*, (ii) picoeukaryotic phytoplankton, which were identified based on the PerCP channel and forward scatter with picoeukaroytes being larger and having higher fluorescence signals than *Prochlorococcus*; and (iii) *Synechococcus* cells, which were identified based on their emission in the phycoerythrin (PE) channel. Samples were then stained with SYBR Green I (Molecular Probes/Thermo Fisher) to a final concentration according to the manufacturer’s instructions and the total microbial population was counted. Data were acquired and processed with FlowJo software. Flow rates were determined several times during each running session, and the average value for a specific run was used for calculating cells per ml.

### Pigment Analysis

Phytoplankton community structure was identified by pigment analysis. Four to 11 L seawater were filtered on Glass fiber filters (25 mm GF/F, Whatman, nominal pore size 0.7 μm). Excess water was removed from the filters by placing their underside on a kimwipe and then transferred to cryovials, stored in liquid nitrogen until arrival at the laboratory, and kept at −80°C until extraction. The collected cells were extracted in 1 ml 100% methanol for 2.5 h at room temperature. Extractions were immediately clarified with syringe filters (Acrodisc CR, 13 mm, 0.2 μm PTFE membranes, Pall Life Sciences) and transferred to UPLC vials. Samples were run on a UPLC and pigments identified based on retention time and spectrum absorbance. Several known standards [DHI Water and Environment Institute, (Hørsholm, Denmark)] were used to ease identification and calculate pigment concentrations (see Supplementary Information for details).

### DNA and RNA Sample Collection and Extraction

Seawater samples were collected from the Niskin bottles into 10 or 20 L polycarbonate carboys. Tubes for water transfer and the carboys were pre-rinsed three times with sample water. Samples for DNA (5–11.5 L) were pre-filtered through 11 and 5 μm nylon filters (Millipore), cells collected on 0.22 μm Sterivex filters (Millipore) and kept in storage buffer (40 mM EDTA, 50 mM Tris pH 8.3, and 0.75 M sucrose). Samples for RNA (0.5–4.5 L, according to volume filtered within 15 min from Niskin bottles being on-deck) were collected without prefiltration, directly on 0.2 μm filters (Supor-200 Membrane Disk Filters, 25 mm; Pall Corporation) to speed the filtration process and obtain enough biomass within a 15 min filtration time. While the lack of prefiltration might affect the perceived community composition, particle-associated bacteria are thought to be a relatively minor fraction of the community (see Supplementary Figure S2 in Mestre et al., 2017). Filters for RNA extraction were preserved in RNA Save (Biological Industries). DNA and RNA samples were stored in liquid nitrogen onboard and kept at −80°C in the laboratory until extraction. Nucleic acids were extracted at the BioRap unit, Faculty of Medicine, Technion, Israel using a semi-automated protocol, which includes manually performed chemical and mechanical cell lysis before the automated steps (see Supplementary Information for details).

### PCR Amplification and Sequencing of 16S rRNA Genes (DNA) and Transcripts (RNA) Samples

Prior to reverse transcription, all RNA samples were tested by PCR for the presence of contaminating DNA. Total RNA was reverse transcribed using the iScript cDNA synthesis kit (Bio-Rad) according to the manufacturer’s instructions. A two-stage “targeted amplicon sequencing” protocol (e.g., Green et al., 2015) was performed to PCR amplify the 16S rRNA gene from cDNA to DNA (see Supplementary Information for a detailed description). The primers used in the first PCR stage consisted of the 16S primer set 515F-Y and 926R (Parada et al., 2016) that targets the variable V4-5 region with common sequence tags added at the 5′ end as described previously (e.g., Moonsamy et al., 2013). The first PCR stage was performed in triplicate, and the three PCR reactions were pooled after validation on 1% agarose gels. Subsequently, a second PCR amplification was performed to prepare libraries. These were pooled after a quality control and sequenced (2 × 250 paired-end reads) using an Illumina MiSeq sequencer. Library preparation and pooling were performed at the DNA Services (DNAS) facility, Research Resources Center (RRC), University of Illinois at Chicago (UIC). MiSeq sequencing was performed at the W.M. Keck Center for Comparative and Functional Genomics at the University of Illinois at Urbana-Champaign (UIUC).

### Sequence Processing

Processing of the sequenced data followed the same pipeline as outlined in Roth Rosenberg et al. (2021). Briefly, after quality control of the obtained pair of fastq files for each sample, paired-end sequencing reads were merged using Flash (version 1.2.11) with the following parameters: max-overlap: 95; min-overlap: 85, with all other parameters set to default (Magoč and Salzberg, 2011). Merged reads were denoised and pre-processed using Dada2 (version 1.1.6; Callahan et al., 2016). Reads were trimmed to 400 nucleotides, while all other parameters were set to default values. Chimeric PCR products were removed using the “tableMethod” parameter set to “consensus.” Taxonomy was assigned to Exact Sequence Variants (ESVs) with the “classify.seqs” command in MOTHUR, the SILVA database (version 128), and an 80% identity cutoff (Schloss et al., 2009). ESVs identified non-prokaryotic, chloroplasts or mitochondria origin were removed. In total, the 56 samples had 2,678,521 quality sequences (range 21,488–75,136 sequences per sample, average: 478301). To avoid bias related to differences in library size, all libraries were rarified to 21,000 reads per sample, using the “rrarify” command implemented in the R Package Vegan (Oksanen et al., 2017). Read data were deposited in the NCBI SRA database under the project number PRJNA548664. Station 4 data are labeled N1200, a change made to ease the reading of the article.

### Sequencing Controls and Variance Among Replicates

Four negative controls from the first PCR were added randomly into the sequencing plates to monitor overall potential for cross contaminations in both PCR and sequencing. These negative control samples averaged 929 reads, compared to samples averaging 50,503 reads. Accordingly, contaminant DNA should poorly compete with sample DNA for amplification.

To assess within station variability and robustness of the observed trends, duplicates for DNA and RNA samples were collected and extracted for samples from station 4 for the summer 2015 cruise, the spring 2015 and 2016 cruises, and for station 1 for the spring 2016 cruise. We chose these samples to have a representation from different seasons, as well as from the most coastal and most offshore stations. Based on their Bray–Curtis dissimilarity, replicates were significantly closer to each other than to other stations from the same cruise (paired Wilcoxon signed-rank test, RNA: *z* = 3.9395 and *p* < 0.001; DNA: *z* = 4.1899 and *p* < 0.001).

### Microbial Community Structure Analysis

The R package vegan version 2.5-7 (Oksanen et al., 2017) was used to calculate alpha diversity parameters (Chao1 richness, Shannon H′ diversity, and inverse Simpson), beta-diversity by pairwise calculation of the Bray–Curtis dissimilarity, and for non-metric multidimensional scaling analysis. Vegan was also used for non-metric multidimensional scaling analysis based on the Bray–Curtis dissimilarity matrix with *k* = 2, 100 runs and 100 iterations. Significance of the grouping factors molecule type (RNA vs. DNA), season (early winter, spring, and summer), and sampling station (stations 1–4) was assessed with the Vegan “adonis” command was used based on Bray-Curtis dissimilarities with 999 permutations.

Correspondence between abiotic and biotic measurements and variation in community composition was examined by variation partitioning analysis and distance-based redundancy analysis. First, the proposed explanatory variables were divided into three matrices: (I) physical: distance from shore, temperature, salinity, and turbidity; (II) nutrients: phosphate, NO_3_ + NO_2_, and silicate; and (III) biotic: total fluorescence as proxy for chlorophyll, picoeukaryote counts, and total cell counts. Variation partitioning calculates the canonical coefficient of determination (adj. *R*^2^), representing the forecasting potential of multiple regression between the response variables (i.e., the ESVs) and explanatory variables (i.e., environmental measurements). Variation partitioning was performed using Vegan “varpart” command. Permutation tests, to validate the significance of the coefficients (adj. *R*^2^), were executed both directly and conditionally (excluding variation that is related to interactions between the different explanatory variable matrices) by calculating distance-based redundancy analysis (dbRDA) using the “dbRDA” command followed by “anova” for the model and the tested variables.

Seasonal and spatial preferences within a season of ESVs were analyzed with the “multipatt” command with 9,999 permutations of the R package indicspecies version 1.7.7 (De Cáceres et al., 2010).

### Statistical Analyses

In addition to statistical tests described above, we used paired Wilcoxon signed-rank tests to test if Bray–Curtis dissimilarity between replicates from the same sample location was lower than between samples from different locations from the same cruise. A Kruskal–Wallis test was used to find significant differences in alpha diversity indices between seasons, followed by Dunn’s *post-hoc* test with Bonferroni correction of value of *p*. Two-tailed Mann–Whitney U tests were used to determine for each season if Bray–Curtis dissimilarity between station 1 and the other three stations was higher than between the three further offshore stations. Spearman correlations were performed to test for correlation between station distance and Bray–Curtis dissimilarity between samples from the same cruise. Spearman correlations were also used to test for correlation between DNA and RNA abundances at the family level. All these tests were performed in PAST version 4.05 (Hammer et al., 2001).

## Results

### Environmental Setting

Satellite (Figure 1) and CTD data (Supplementary Figure S1) indicated that our sampling times occurred in three distinct stages of the annual mixing and stratification cycle in the EMS (Kress and Herut, 2001; Ben Ezra et al., 2021): (i) the stratified water column in summer, (ii) early mixing and phytoplankton bloom in early winter, and (iii) the declining surface water phytoplankton bloom in spring after its usual peak in January/February (Reich et al., 2021; Roth Rosenberg et al., 2021). Pigment data indicated that these stages differed in their phytoplankton community. Early winter samples were dominated by haptophytes of either Phaeocystaceae or members of the Prymnesiaceae and Isochrysidaceae families. Spring samples were characterized by peak abundances of *Prochlorococcus* (Supplementary Figure S5D; Supplementary Table S6), and summer samples had the lowest *Synechococcus* abundances at the sampling depth at stations 2–4 (Supplementary Figure S5C; Supplementary Table S6; Supplementary Information includes further details on the pigment analysis and results).

The ultra-oligotrophic status of the Levantine basin with extremely low nutrient concentrations in its surface waters (Krom et al., 2005; Ben Ezra et al., 2021; Reich et al., 2021) was consistently observed in our samples (Supplementary Tables S2 and S3). Phosphate concentrations were near or below our practical detection limit (40 nM) in almost all cruises. Nitrate + nitrite concentrations for these samples ranged from <50 to 240 nM at the most offshore station and from <50 to 690 nM at the most coastal station. At the most coastal station, winter samples were clearly higher in nitrate + nitrite concentrations than spring and summer samples, with no clear difference between the latter two. At the further offshore stations, no systematic difference in nitrate + nitrite could be found between winter and spring samples, but summer samples were always at or below our detection limit. In early winter nitrite + nitrate concentrations decreased from station 1 toward the offshore stations, whereas in spring, they increased from station 1 to station 3 before decreasing again toward station 4, the most offshore station. Additional analysis of fresh (unfrozen) samples provided a lower detection limit (Ben Ezra et al., 2021) and was carried out in the summer 2016 cruise (Supplementary Table S3), one of the most nutrient depleted sampling periods. These data show that, although most of our nutrient measurements fell below the detection limit, the sampling transect was able to capture a nutrient gradient impacted by the proximity to the shore.

Other environmental parameters varied with different degrees of change. For instance, surface seawater temperature varied significantly with the seasons, with the lowest temperatures observed in spring (17.7°C–18.6°C) and highest in summer (26.2°C–28.7°C). However, the seasonal changes in salinity were small (<0.7 PSU from lowest to highest value; Supplementary Table S2), suggesting minimal water mass intrusions from other locations.

Overall, environmental parameters changed more between seasons than between station locations within a cruise. The average difference between minimal and maximal temperature across seasons at a sampling site was 10.7°C compared to only 0.3°C between sites within a given cruise. The same could be observed for salinity (0.59 vs. 0.14 PSU), fluorescence (0.36 vs. 0.08), and turbidity (4.4 vs. 0.54 FTU). Within the nutrient data, these differences were smaller. Nitrate + nitrite differed on average 0.44 μmol/L between the lowest and highest value at a given site between seasons compared to an average difference of 0.3 μmol/L between the highest and lowest value within a cruise. For silicate, this was 0.62 μmol/L compared to 0.54 μmol/L. We did not compute these differences for phosphate as values were frequently below the detection limit.

Along the transect the collected environmental data differed the most between station 1, the shallowest and most coastal sampling site (16 km from shore), and the other three more offshore stations (25–50 km from shore). Station 1 often had higher nutrient concentrations (e.g., silicate in spring and nitrite + nitrate in early winter; Supplementary Tables S2 and S3) and chlorophyll concentrations (Figure 1) throughout the year. The station closest to the shore also differed from the rest in temperature (higher in early winter and spring), salinity (lower in summer), fluorescence (higher in early winter), and turbidity (higher in early winter and summer 2016, lower in summer 2015; Supplementary Table S5; Supplementary Figure S1).

### Microbial Community Composition

**Figure 2:**
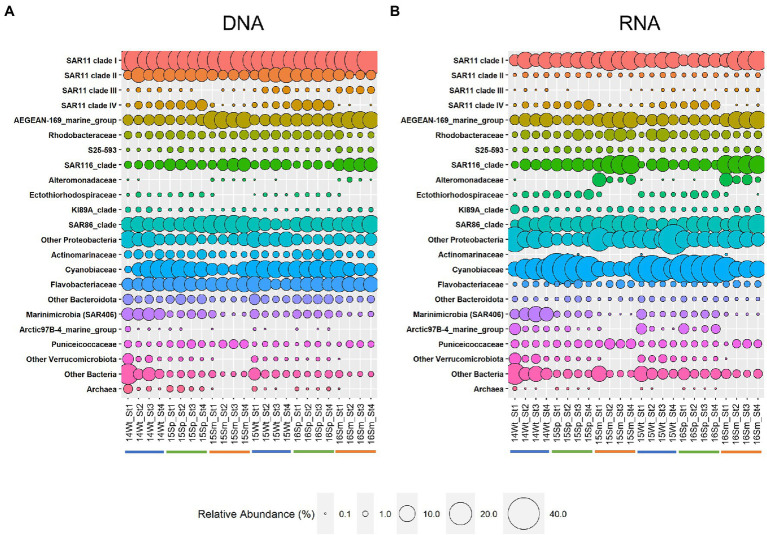
Relative abundance at the family level in DNA **(A)** and RNA **(B)** samples. Families with >1% total reads in either DNA or RNA are shown. Families below this threshold are summarized at the phylum level except for the SAR11 clade III. Phyla with <1% of reads in either DNA or RNA are summarized at the domain level. Occurrences of less than 0.1% in a sample are indicated as missing. Sample code: last two digits of collection year (20XX), season (Wt, early winter; Sp, spring; and Sm, summer), and station (St1-4). Color bars under the sample name indicate seasons: blue, winter; green, spring; and orange, summer.

The best-represented microbial groups were recurrently identified throughout the study. However, their proportions and levels of activity (retrieved as community 16Sr RNA) showed some variation at different sampling locations and times (Figure 2). Bacteria dominated the microbial community at the 10 m sampling depth throughout the year at all stations. After subsampling, they made up 99.7% of all reads (3,240 ESVs) compared to 0.3% of archaeal reads (39 ESVs). Most bacterial reads (82.6%) belonged to one of three classes: Alphaproteobacteria (47.0% of all reads), Gammaproteobacteria (18.5%), and Cyanobacteria (17.1%). The Alphaproteobacteria were dominated by the SAR11 clade (57.4% of the Alphaproteobacteria), the Cyanobacteriia by *Prochlorococcus* and *Synechococcus* (together 94.1% of the Cyanobacteriia), and the Gammaproteobacteria by the SAR86 clade (53.3% of the Gammaproteobacteria). Only four other classes contributed more than 1% of total reads: Bacteroidia (6.0%), Verrucomicrobia (3.3%), Marinimicrobia (SAR406 clade) (2.4%), and Acidimicrobiia (1.4%). The archaea were dominated by marine group II Thermoplasmatota (68.4% of all archaeal reads), followed by Thermoplasmatota unidentified at the family level (21.0%) and Nitrosopumilaceae Thaumarchaeota (10.6%). Figure 2 shows the relative abundance of the 18 families representing >1% of either the total DNA (Figure 2A) or RNA (Figure 2B) reads.

**Figure 3:**
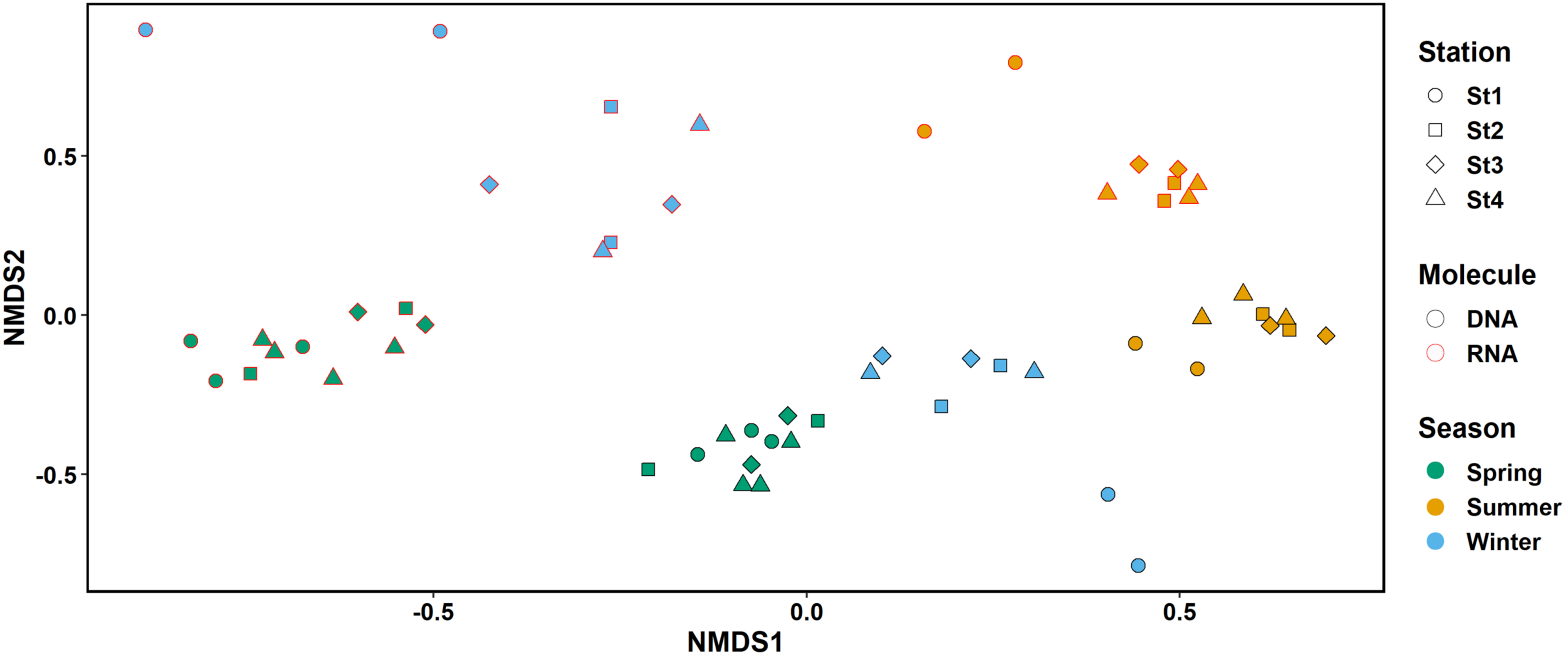
NMDS plot of the RNA (red border) and DNA (black border) samples. Colors represent seasons: spring, green; summer, orange; and early winter, blue. Shapes represent different stations. *K* = 2, stress: 0.132.

Comparison of the microbial community structure using 16S rRNA allowed us to further identify the most active microbial groups in these communities. For 15 of the 18 families shown in Figure 2, we found a significant positive correlation (Spearman correlation, *r* > 0.6, *p* < 0.05) between relative abundance in DNA and corresponding RNA samples (Supplementary Table S7), indicating a strong link between presence and activity. However, the average RNA:DNA ratios differed between family-level taxonomic groups (e.g., 0.36 ± 0.09 for SAR11 clade I and 2.48 ± 0.91 for Cyanobiaceae) and the composition of resident (DNA) and active (RNA) microbial communities clearly differed (Figure 2), as supported by non-metric multidimensional scaling analysis (NMDS; Figure 3) and Adonis test (*F* = 12.9, *R*^2^ = 0.219, *p* < 0.001). Given this difference, we performed all subsequent analyses of microbial community composition (DNA) and activity (RNA) separately.

### Season Had a Larger Effect on Microbial Structure and Activity Than Spatial Location

Sampling season had a clear impact on both the microbial community structure (DNA) and activity (RNA; Figure 3). The effect was significant and explained 54.9 and 55.9% of the variability in RNA and DNA samples, respectively. In contrast, the effect of station location across the coastal to offshore transect on the observed variability was small (8.7% and 7.6% in RNA and DNA samples, respectively) and not significant (Adonis test, DNA: season *R*^2^ = 0.55944, station *R*^2^ = 0.07566; RNA: season *R*^2^ = 0.54948, station *R*^2^ = 0.08692; *p* < 0.001 for season and *p* > 0.05 for station in both analyses, see details in Supplementary Table S8).

In addition to microbial community structure, the overall microbial diversity also changed significantly with season (Supplementary Figure S3). For DNA samples, the three alpha diversity indices (Shannon H, inverse Simpson, Chao1) indicated the lowest diversity in summer (Kruskal–Wallis test, *p* < 0.001 followed by Bonferroni corrected Dunn’s *post-hoc* test *p* < 0.05), when the system was most oligotrophic, with no difference between winter and spring samples. All three indices also varied significantly seasonally in the RNA samples (Kruskal–Wallis test, *p* < 0.01, followed by Bonferroni corrected Dunn’s *post-hoc* test *p* < 0.05), but the pattern was less clear.

Given the particularly high abundance of members of the SAR11 group in the EMS in our samples (average of 36.9% of DNA reads) and in previous studies (e.g., Dubinsky et al., 2017), we further analyzed the seasonality of its different clades. The SAR11 clade Ia was the dominant SAR11 clade contributing on average 61.3% of all SAR11 DNA reads (range 41.0%–90.2%). It was more abundant during the summer months than in spring and winter of the two sampling years (Supplementary Figure S4). In contrast, an opposite trend was observed for SAR11 clades Ib, II, and IV, for which relative abundances were lowest during the summer, especially at the more oligotrophic stations 2–4 (Supplementary Figure S4). These data support the notion that different SAR11 clades potentially occupy distinct ecological niches in this ultra-oligotrophic environment.

Other relatively abundant groups with clear seasonal patterns included the AEGEAN-169 marine group of the Alphaproteobacteria family Rhodospirillaceae as well as the SAR86 and SAR116 clades (all most abundant in summer; Figure 2). Within the family Cyanobiaceae, *Prochlorococcus* showed a clear peak in spring, whereas no clear pattern was seen for *Synechococcus* (Supplementary Figure S5).

### Effects of Environmental Parameters on the Microbial Community Structure and Activity

To better understand the influence of different parameters on microbial structure and activity, we examined three environmental data matrices: (i) physical data (distance from shore, temperature, salinity, and turbidity), (ii) biological data (fluorescence as a proxy for chlorophyll, picoeukaryotic phytoplankton, and total microbial cell number), and (iii) nutrient data (phosphate, nitrate + nitrite, and silicate). Together, the three matrices explained about 67% of the observed variability in both the microbial structure (DNA samples) and the microbial activity (RNA samples). Partition analysis indicated that in both analyses, the physical data matrix explained most of the variability (44.5% and 49.4% for DNA and RNA, respectively), followed by the biological data matrix (30.2% and 28.3% for DNA and RNA, respectively) and the nutrient data matrix (17.1% and 13.0% for DNA and RNA, respectively). The physical data matrix also had the largest percent of variability not explained by any of the other two matrixes (DNA: 22.6%; RNA: 24.2%), whereas the nutrient matrix had the lowest (DNA: 0.4%; RNA: 1.1%; Supplementary Figure S6). This suggested that particular environmental factors within these data groups might be responsible for the different levels of prediction. To identify those key parameters, we then performed a distance-based redundancy analysis. As in the partition analysis, the physical matrix was able to explain the largest amount of variance, followed by the biological matrix and the nutrient matrix. Yet, the percent variation explained by the nutrient matrix was not significant after correcting for the other two matrices. Overall, environmental factors that tended to change with season, such as temperature and salinity from the physical matrix, and fluorescence and total cell count from the biological matrix were factors with a significant level of prediction in the DNA and RNA analysis (both in the unconditioned and conditioned analysis, *p* < 0.05, Supplementary Figure S6).

### Seasonality at the ESV Level

We used an indicator species analysis to identify ESVs with seasonal preferences. Significant association with one or two seasons was found in 299 ESVs in the DNA samples representing 77.5% of all DNA reads (Supplementary File 1—sheet 2). In the RNA samples, 386 ESVs representing 67.3% of all RNA reads had significant associations with one or two seasons (Supplementary File 1—sheet 3). When we compared the 202 ESVs that had significant seasonal preferences in both the DNA and RNA analyses, 181 ESVs had the same seasonal preferences in both analyses and another 20 ESVs had an additional season in one of the analyses.

The indicator analyses identified several taxonomic groups favoring specific seasons (see Supplementary File 1—sheets 2 for DNA and sheet 3 for RNA indicator ESVs): for example, all indicator Alteromonadaceae ESVs were specific for summer; several taxonomic groups were (mostly) indicator for spring (e.g., most ESVs of *Prochlorochoccus*, SAR11 clade Ib and II, all ESVs of Actinomarinaceae, and SAR11 clade IV) and some for early winter (e.g., most indicator ESVs of the Chloroflexi clade SAR202, the Deltaproteobacteria clade SAR324, and the Gammaproteobacteria clade OM182). However, most taxonomic groups had indicator species from several seasons: for example, most ESVs from SAR11 clade Ia were either associated with summer or spring at the DNA level. The Marinimicrobia clade SAR406 had ESVs indicator for spring and early winter, but not for summer. Likewise, the significant *Synechococcus* ESVs associated with spring, spring and early winter, or early winter, but not summer. ESVs from the SAR86 clade associated with summer, spring, and early winter at the DNA level, with most of the abundant ESVs being indicators for summer.

To analyze which ESVs dominate the community and whether dominant and rare ESVs show different patterns, we classified ESVs into four categories broadly following the categories in Auladell et al. (2021): dominant (present in ≥75% of the samples with ≥1% relative abundance), rare (always <1%), intermediate persistent (present in ≥75% of samples), and intermediate fluctuating (present in <75% of samples). Despite the large influence of season on the microbial community, the microbial community structure was dominated by only a few bacterial ESVs throughout the year (Table 1). The eight dominant ESVs in the DNA data made up together on average 38.5% of the community and were especially dominant in summer when they averaged 48.4%. Four of these ESVs belonged to SAR11 clade Ia and one each belonged to SAR11 clade II, AEGEAN-marine group, *Ca.* Actinomarina and *Synechococcus*. Especially noteworthy was ESV000001, a SAR11 clade Ia strain, which was the most abundant ESV in all but three DNA samples and made up on average 11.6% of the community.

In the RNA data, only five ESVs fell into the dominant group: three SAR11 clade Ia, a *Synechococcus* ESV—all of which were also dominant in the DNA data—and a SAR86 ESV. Together these ESVs represented on average 19% of the RNA community data (Table 1). The *Synechococcus* ESV (ESV000003) was the most active one across most samples and represented on average 8.7% of the RNA reads per sample. The only other ESV contributing always more than 1% to the RNA reads was ESV0000001 of SAR11 clade Ia, which was the most abundant ESV in the DNA data. ESV0000002, a *Prochlorococcus* strain, that was in the intermediate persistent group, had the highest overall abundance (32.6% of the RNA reads), was among the top 5 most abundant ESVs in 12 of the 24 samples (average RNA relative abundance 8.69%), and had a clear seasonality. Together these three strains represented on average 22% and maximal 40.9% of the RNA reads.

### Spatial Effects: Microbial Communities at Coastal Station 1 Differ From Those at Offshore Stations in Summer and Early Winter, but Not in Spring

Distance from shore was not a significant factor when analyzing the effect of physical parameters on the community (Supplementary Figure S6). We also found no significant correlation of distance between stations and Bray–Curtis dissimilarity within cruises (neither when all cruises were analyzed together or cruises were split by season; Spearman correlation, *p* > 0.05). However, samples from the most coastal station 1 grouped apart from the samples of the more offshore stations 2–4 in the NMDS plot in early winter and summer, but not in spring (Figure 3).

**Figure 4:**
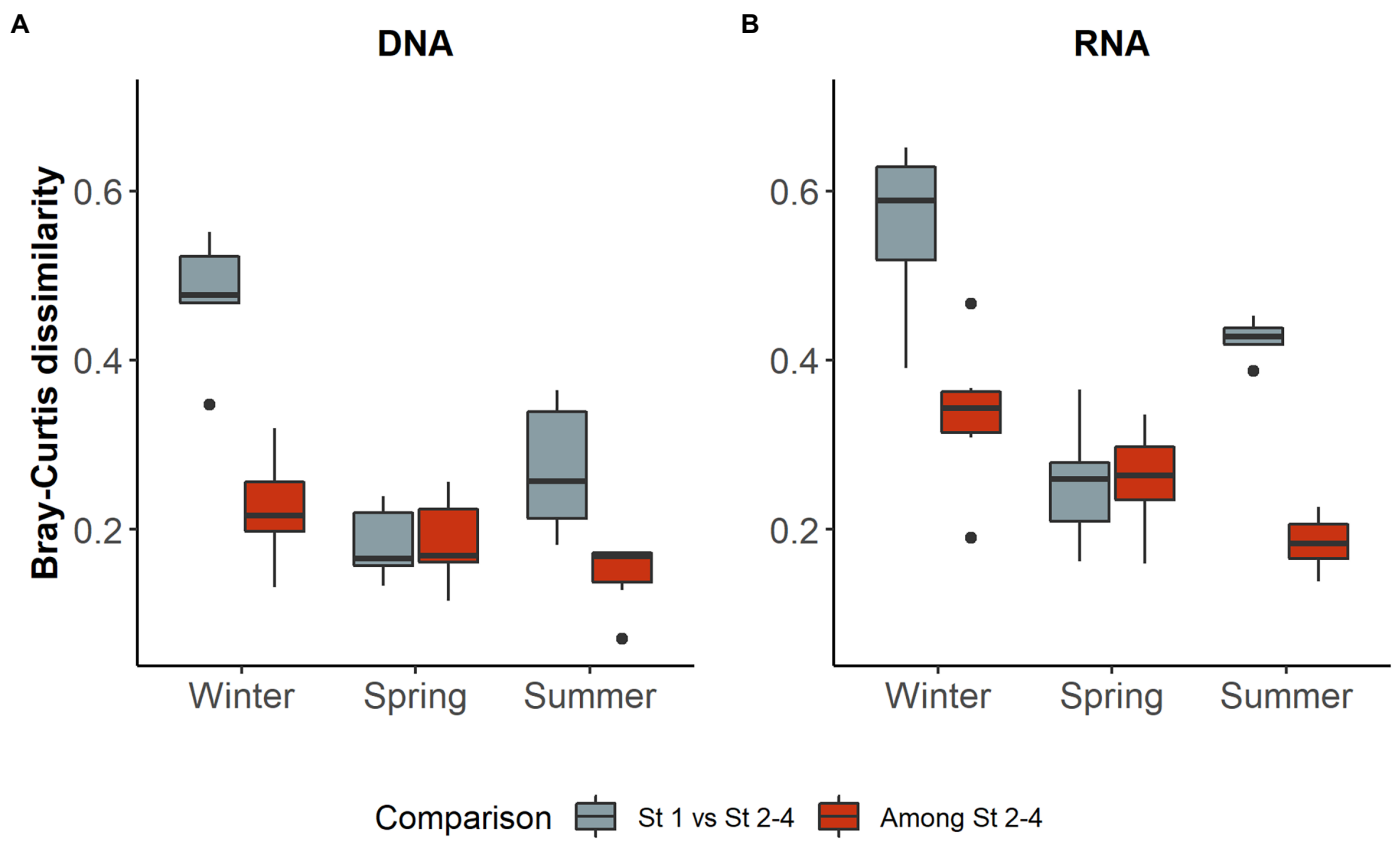
Bray–Curtis dissimilarity in different seasons of **(A)** DNA and **(B)** RNA samples in comparisons including and excluding station 1. Only dissimilarities between stations from the same cruise were used. In both sample types, comparisons involving station 1 differed significantly from those among the other stations in early winter and summer (Mann–Whitney U test, two-tailed, winter: RNA *z* = 2.6421, *p* < 0.005; DNA: *z* = 2.8022 *p* < 0.005; summer: RNA *z* = −2.8022, *p* < 0.005; DNA: *z* = −2.8022, *p* < 0.005), but not in spring (RNA: *z* = −0.080064, *p* = 0.937; DNA: *z* = 0.24019, *p* = 0.818).

As station 1 also differed in environmental parameters from the three more offshore stations (see environmental settings above), we tested if this difference could be reflected in the microbial community structure (DNA) and activity (RNA). In both early winter and summer within cruises, Bray-Curtis dissimilarities were significantly higher between station 1 and the other three stations than among the three offshore stations, while no difference was observed in spring (Figure 4).

Indicator species analyses performed for each season separately found 92 ESVs in the DNA data exhibiting a spatial preference (either station 1 or station 2–4), of which 13 showed significant association in multiple seasons with consistent spatial preference (details for each ESV are given in Supplementary File 1 sheets 4). Similar results were found in the active community as 129 ESVs showed a significant spatial preference in the RNA samples (Supplementary File 1 sheet 5).

Overall, several taxonomic groups showed a spatial preference among their indicator ESVs (Supplementary File 1 sheets 4 and 5). Indicator ESVs of archaea, SAR11 clade IV, SAR202 Chloroflexiota, Bdellovibrionaceae clade OM27, Marinimicrobiota SAR406, Planctomycota, and most Verrucomicrobiota (except ESVs of *Lentimonas* and R76-B128) were associated with the coastal station 1. In contrast, several ESVs of the SAR11 clade I and most of SAR11 clade II, SAR86, and SAR116 were indicators for the offshore stations 2–4 (Supplementary File 1 sheets 4 and 5). Within Cyanobacteria *Cyanobium* ESVs were associated with station 1, while *Prochlorococcus* and *Synechococcus* ESVs were associated with the offshore stations 2–4.

## Discussion

The ultra-oligotrophic nature of the EMS is reflected in the microbial community structure of this coastal-offshore transect. Typical oligotrophic groups (e.g., SAR11) dominated throughout the year confirming previous snapshot studies of the EMS (Feingersch et al., 2010; Dubinsky et al., 2017) and implying that the analyzed part of the EMS is a relatively homogeneous ultra-oligotrophic system. Hence, our results support the nutrient analysis based notion by Powley et al. (2017) that the EMS, although an inland sea, behaves in a similar way to open ocean gyres. The observed abundance of the dominant group, SAR11, throughout the year is consistent with data reported for oligotrophic ocean gyres (Carlson et al., 2009; Eiler et al., 2009; Morris et al., 2012; West et al., 2016) though high SAR11 abundances have also been reported from surface waters of offshore stations in the oligotrophic Southern Adriatic Sea (Korlević et al., 2015; see Supplementary Table S9 for a comparison of Mediterranean and open ocean abundances). SAR11 relative abundances at our most coastal station 1 were higher (36%–42% of DNA reads in spring and summer) than those found at coastal stations in other parts of the Mediterranean Sea, including the Northwestern Mediterranean, Southern Mediterranean, and the Adriatic Sea (Alonso-Sáez et al., 2007; Tinta et al., 2015; Quéméneur et al., 2020; Auladell et al., 2021; see Supplementary Table S9), highlighting the overall more oligotrophic status of the South Eastern Levantine basin even close to the coast.

In our study, season affected the microbial community structure and activity much more than spatial location along the coastal-to-offshore transect. This differs from other studies that analyzed spatial–temporal community changes in coastal-offshore transects. Fortunato et al. (2011) described that samples clustered into five groups based on their microbial community composition that corresponded to a stable spatial separation between groups of sampling sites from the Columbia river to the offshore throughout the year. Seasonal differences were observed only within site groups. This could be explained by the salinity gradient along the transect, as indicated by the high correlation with salinity (Fortunato et al., 2011). In the absence of particular salinity gradients, Wang et al. (2019) found a strong influence of sample location on microbial community structure along a transect from the coastal PICO station out to the Sargasso Sea. Yet, a seasonal effect was seen in the change of clustering of shelf stations and the strong influence of temperature on the microbial community structure. A key difference between our study and the one by Wang et al. (2019) is the strength of the environmental gradient between the coastal and offshore stations and the overall productivity of the coastal area. Primary productivity levels at the PICO station were usually above 1,000 mg C m^−3^ d^−1^, which is more than an order of magnitude higher than that of their most offshore station (Wang et al., 2019). In our EMS study area, coastal primary production averaged around three with a maximum of 12 mg C m^−3^ d^−1^ (Raveh et al., 2015) compared to 1–3 mg C m^−3^ d^−1^ measured at offshore surface waters (Reich et al., 2021), a difference of only about 3–4 fold. The spatial effects on microbial community structure between the most coastal station 1 and the offshore stations were detected in early winter and summer, but not in spring, when coastal and offshore communities were highly similar. This is in line with an earlier study based on chlorophyll and temperature, describing a clear delineation between coastal and offshore waters from late spring to early winter, which then diffused from winter to early spring (Berman et al., 1986).

Indicator analysis showed that most significant ESVs from typical oligotrophic groups such as SAR11, SAR86, SAR116, *Prochlorococcus*, and *Synechococcus* were associated with the offshore stations 2–4. Most significant indicator ESVs for the coastal station 1 belonged to more copiotrophic groups (e.g., ESVs of Flavobacteriales, the Gammaproteobacteria OM60/NOR5, the OM27 clade, Planctomycetes, and Verrucomicrobiae) or to groups typical of deeper waters (e.g., ESVs of the Marinimicrobia SAR406 clade and marine group II archaea). Most of these spatial indicator ESVs were significant in their spatial preference in either early winter (70 ESVs in the DNA and 77 ESVs in the RNA analysis, respectively) or summer (28 and 40 ESVs, respectively) when there is a sharper separation between coastal and offshore waters in the EMS compared to spring (Berman et al., 1986). Hence although the gradient in nutrients and productivity is small between coastal and offshore waters in the EMS, it was still detected in the spatial preference of certain microbial groups.

Profound and predictable seasonal shifts in marine surface water microbial communities have been shown in several time-series studies (e.g., Fuhrman et al., 2006, 2015; Ward et al., 2017; Galand et al., 2018) and, as in our case, temperature has been frequently identified as a main environmental driver (e.g., Sunagawa et al., 2015; Lucas et al., 2016; Ward et al., 2017). Salinity, like temperature, is often an indicator for seasonal changes in hydrography, and it is often identified as a seasonal driver of marine surface water microbial communities (e.g., Fuhrman et al., 2006; Ward et al., 2017). Despite the fact that seasonality is expected to strongly affect primary productivity and nutrient availability in the EMS (Kress and Herut, 2001; Krom et al., 2014; Raveh et al., 2015), our measured nutrient data explained the seasonal shifts in microbial communities to a lesser extent than the measured physical parameters. It is possible that the effect of nutrients was not detected as several measurements, especially phosphate, were below the detection limit of our analysis method. However, recent seasonal high sensitivity nutrient measurements using fresh unfrozen samples (such as those analyzed only in the July 2016 cruise) at an offshore station close to our sampling sites indicated that phosphate is close to the detection limit of <6 nM throughout the year with little changes, while nitrite + nitrate showed seasonality from a peak of 300 to 500 nM in winter to less than 50 nM in summer (Ben Ezra et al., 2021). Seasonality thus might be caused by difference in nitrate + nitrite availability, whereas a general tolerance of low phosphorus concentrations is needed throughout the year. Indeed, this general adaptation of P limitation has been attributed to observed differences in microbial community structures between the Western and Eastern basin of the Mediterranean Sea and the dominance of different SAR11 ESVs (Sebastián et al., 2021). Overall, our measured parameters explained around two-thirds of the observed variability in the microbial community both at the DNA and the RNA level. Therefore, it is likely that other factors not measured in this study were responsible for the unexplained variability. These may include UV radiation, which is known to penetrate deep into the clear waters of the EMS with UV doses among the highest of all oceans (Tedetti and Sempéré, 2006; Smyth, 2011), irradiance, a factor known for structuring surface water microbial communities in the subtropical North Pacific Gyre station ALOHA (Bryant et al., 2016), and organic nutrients such as phytoplankton derived organic carbon (Camarena-Gómez et al., 2018).

The recurrent shifts in microbial structure and activity followed typical seasonal patterns. Physical mixing of the water column in early winter seemed to be responsible for a “resetting” of the microbial ecosystem from a low diversity state in summer, when the microbial community in the EMS is nutrient depleted (Thingstad et al., 2005; Tanaka et al., 2011; Ben Ezra et al., 2021), to a high diversity state in winter, as previously proposed for the Northwestern Mediterranean Sea (Salter et al., 2015; Auladell et al., 2021). Our two early winter cruises showed differences in some environmental parameters such as temperature and fluorescence (Supplementary Table S5) pointing to different mixing conditions. Nevertheless, the microbial communities were similar as samples grouped next to each other in the nMDS plot (Figure 3) indicating their intermediate stage between the stratified and fully mixed conditions. Mixing of the water column leads to an increased availability of nutrients at the surface, allowing once rare microbes to grow and thrive. In addition, microbes from deeper layers are brought up and might repopulate and grow in the now nutrient-enriched surface waters, as observed in the Western Mediterranean Sea (Haro-Moreno et al., 2018). These “upwelled” microbes might interact with surface water microbes and affect the microbial community as shown in recent mesocosm experiments performed in the EMS (Hazan et al., 2018). Potential candidates of “upwelled” microbes in our study include SAR202 and SAR406 groups that are typical of deeper water (Roth Rosenberg et al., 2021; Sebastián et al., 2021) and were more abundant in early winter and spring than in summer when the water column is stratified. Interestingly, SAR202 and SAR406 ESVs were present not only in the 16S rRNA gene but also in the transcript data (amplicons obtained on cDNA template) collected at the same station and cruise, suggesting that these upwelled taxa remained active in the surface waters. In the EMS, phytoplankton is typically dominated by nano- to micro-sized organisms in winter and picophytoplankton in summer (Raveh et al., 2015). Our pigment data are in line with this observation, as pigments characteristic of haptophytes (19′-hexanoyloxyfucoxanthin, 19′-butanoylfucoxanthin, and fucoxanthin) showed a clear seasonal trend with their highest concentrations in early winter samples. Seasonality in heterotrophic bacteria might thus reflect changes in phytoplankton composition, affecting microbial community structure through the different types of organic carbon produced (Camarena-Gómez et al., 2018).

Recurrent annual microbial patterns can also be observed at the level of closely related strains within specific taxonomic groups that have different seasonal preferences, as found both in this study and at the coastal PICO site (Ward et al., 2017). The seasonal preference is likely due to differences in their genomic makeup, resulting in different ecotypes thriving under different environmental conditions. A clear example is the dominant SAR11 clade. While within clade Ia, we found different ESV preferring summer, winter, and spring, the main seasonal differences observed, were related to seasonal preferences at the SAR11 clade level. These followed the general patterns found at other ocean stations, such as the Mola station in the Northwestern Mediterranean (Salter et al., 2015) and the BATS station in the Sargasso sea (Carlson et al., 2009; Vergin et al., 2013) with some differences. Similar to Mola and in contrast to BATS, in the EMS clade Ib did not replace clade Ia as the dominant clade in spring, though most SAR11 clade Ib ESVs with seasonal preferences were associated with spring. In contrast to Mola, we found a substantial amount of SAR11 clade IV in the EMS, which followed the seasonal pattern described at BATS (Vergin et al., 2013). In a similar way to the SAR11 clade, the dominant indicator ESVs of the SAR86 clade were associated with summer, when nutrients in the EMS are lowest. This is in line with patterns observed at HOT and BATS stations and with the notion of SAR86 being a clade adapted to oligotrophic conditions (Giovannoni and Vergin, 2012). The presence of SAR86 ESVs associated with other seasons suggests the presence of multiple SAR86 ecotypes, as recently predicted based on genomic data (Hoarfrost et al., 2020). Differences in seasonal preference were also found for the main picocyanobacterial genera: *Prochlorococcus* and *Synechococcus.* With one exception, all significant *Prochlorococcus* ESVs were associated with spring, whereas *Synechococcus* ESVs were associated with early winter, spring and early winter, or spring only. *Prochlorococcus* has been shown to be more vulnerable to light and UV irradiance-induced stress than *Synechoccocus* (Mella-Flores et al., 2012), which might explain the preference for spring at the sampling depth of 10 m at this time of the year. Roth Rosenberg et al. (2021) showed that overall *Prochlorococcus* abundance in the study area followed those observed at BATS with peak abundances at the deep chlorophyll maximum in summer.

Overall, our analysis confirms some recurrent observations typical of oligotrophic surface waters. Yet at the same time, it provides a unique perspective on how some particular characteristics of the EMS may affect microbial community structure. Seasonal shifts in microbial communities are linked to functional differences between microbes that enable them to adapt to the changing environment (Galand et al., 2018; Haro-Moreno et al., 2018). Here, we provide a detailed analysis of spatial and temporal changes of microbial communities in the Southeastern Levantine basin. These changes can have a strong impact on biogeochemical cycles yet understanding how the observed microbial community shifts may affect biogeochemical cycles will require metagenomic and metatranscriptomic studies, as well as rate measurements related to these cycles. Despite the strong influence of season on the microbial community structure, the community was dominated by the same few ESVs throughout the year. How these few ESVs can persist over the study period of 2 year remains an open question. Future isolation and characterization of some of these relevant strains will be key to further identify the specific ecological niches they occupy, to determine their level of microdiversity not detected by our sequenced region, and to assess their overall impact in this extreme ecosystem.

## Data Availability Statement

The original contributions presented in the study are included in the article/Supplementary Material; further inquiries can be directed to the corresponding author.

## Author Contributions

MH and LS planed the study. Sampling was conducted by MH, DR, IB, KS, DS, and LS. RL and YL analyzed the satellite data. DA performed the molecular work. Cell counts were conducted by MH and DA. Pigments were analyzed by DR and DS. MH and ML performed the bioinformatic analysis. MDK performed the nutrients analyses. Data interpretation was done by MH, LS, LG-C, MDK, and DS. All authors contributed to the article and approved the submitted version.

## Funding

This study was funded by the Israel Science Foundation grant (ISF #1243/16) to LS and from the United States-Israel Binational Science Foundation (BSF, no. 2019612 to LS) and the United States National Science Foundation (NSF, OCE1924464 to LG-C). The seasonal cruises were supported by funding from the Leon H. Charney School of Marine Sciences (Haifa University, Israel). MH was supported by an Inter-Institutional post-doctoral fellowship from the Haifa University and a Helmsley Trust fellowship.

## Conflict of Interest

The authors declare that the research was conducted in the absence of any commercial or financial relationships that could be construed as a potential conflict of interest.

## Publisher’s Note

All claims expressed in this article are solely those of the authors and do not necessarily represent those of their affiliated organizations, or those of the publisher, the editors and the reviewers. Any product that may be evaluated in this article, or claim that may be made by its manufacturer, is not guaranteed or endorsed by the publisher.

## Footnotes

^1^ http://odv.awi.de

^2^ http://marine.copernicus.eu/services-portfolio/access-toproducts/
