# Supplementary material for: Spatiotemporal Variation of Microbial Communities in the Ultra-Oligotrophic Eastern Mediterranean Sea
Source: Front Microbiol. 2022 Apr 7;13:867694. doi: 10.3389/fmicb.2022.867694 (PMC9022036; doi:10.3389/fmicb.2022.867694)
Supplement: Supplementary file 3 [file Data_Sheet_3.PDF]

Supplementary figures and tables

For the article:

Spatiotemporal variation of microbial communities in the ultraoligotrophic Eastern Mediterranean Sea

Authors:

Markus Haber[1,2], Dalit Roth Rosenberg[1], Maya Lalzar[3], Ilia Burgsdorf[1], Kumar Saurav[1], Regina Lionheart[4], Yoav Lehahn[4], Dikla Aharonovich[1], Laura Gomez-Consarnau[5,6], Daniel Sher[1], Michael D. Krom[1,7], Laura Steindler[1]

Authors' affiliations:

[1] Marine Microbiology Lab, Department of Marine Biology, Leon H. Charney School of Marine Sciences, Haifa University

[2] Department of Aquatic Microbial Ecology, Institute of Hydrobiology, Biology Centre CAS, Czech Republic

[3] Bioinformatics Service Unit, University of Haifa

[4] The Dr. Moses Strauss Department of Marine Geosciences, Leon H. Charney School of Marine Sciences, Haifa University

[5] Department of Biological Sciences, University of Southern California, Los Angeles, USA

[6] Department of Biological Oceanography, Centro de Investigación Científica y de Educación Superior de Ensenada, BC, Mexico

[7] Morris Kahn Marine Research Station, Environmental Geochemistry Lab., Leon H. Charney School of Marine Sciences, Haifa University

Supplementary Figure S1 A: Temperature - depth (up to 500 m) profiles of the transect based on CTD data. All CTD plots were obtained with odv4 (Version 4.7.4) (Schlitzer, R. 2015. Ocean Data View. http://odv.awi.de). Data were stretched by weighted-average gridding with automatic scale length. Bad estimates were hidden, color shading enabled and a quality limit of 5.0 applied. The international temperature scale of 1990 (ITS-90) was used. Contours indicate increments of 1 °C. Stations are indicated by vertical lines, order from left to right: Station 1, 1A, 2, 2B, 3, and 4. Color scale is the same for all plots. X-axis indicates distance from shore.

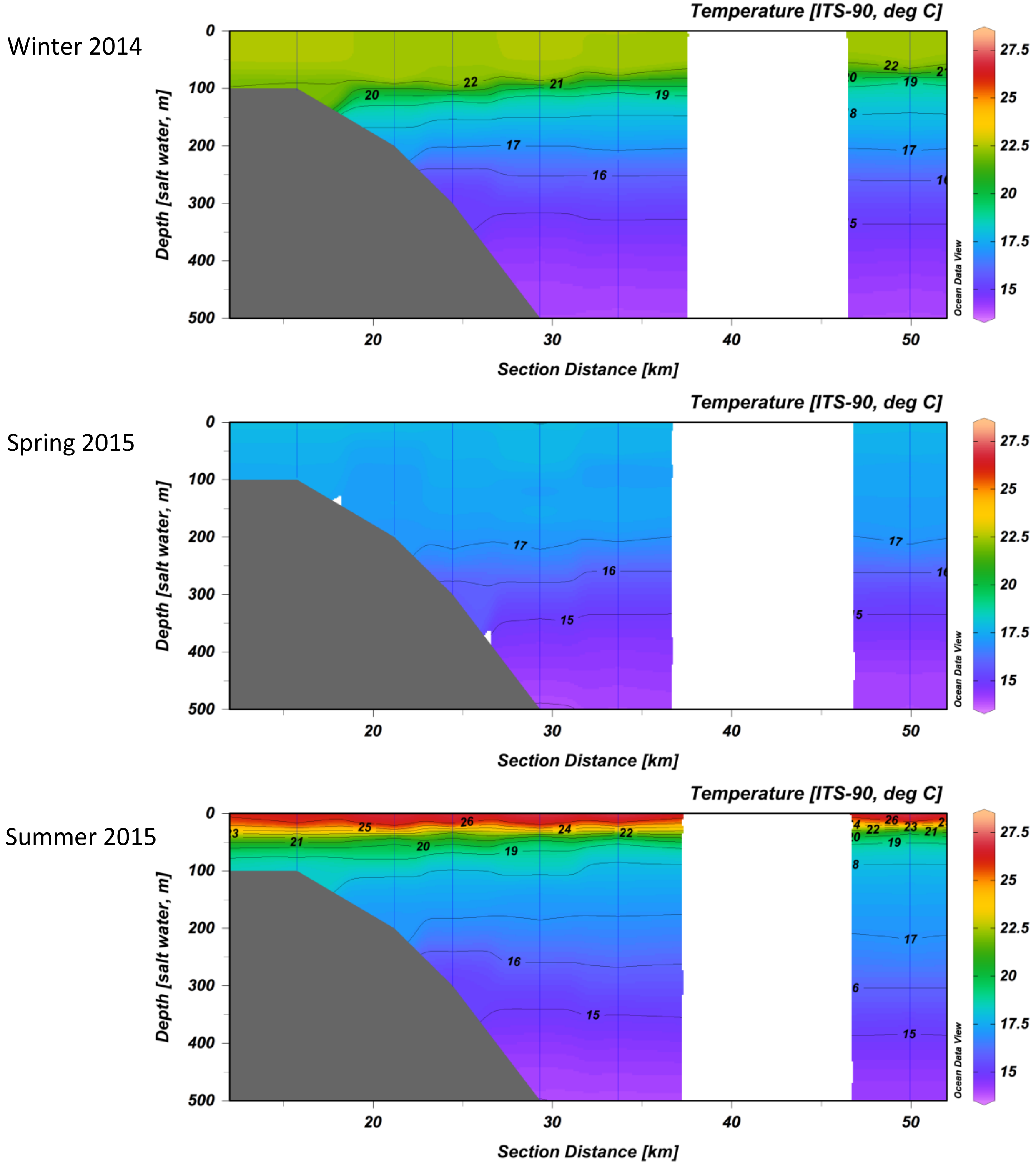

Supplementary Figure S1 A: continued.

Winter 2015

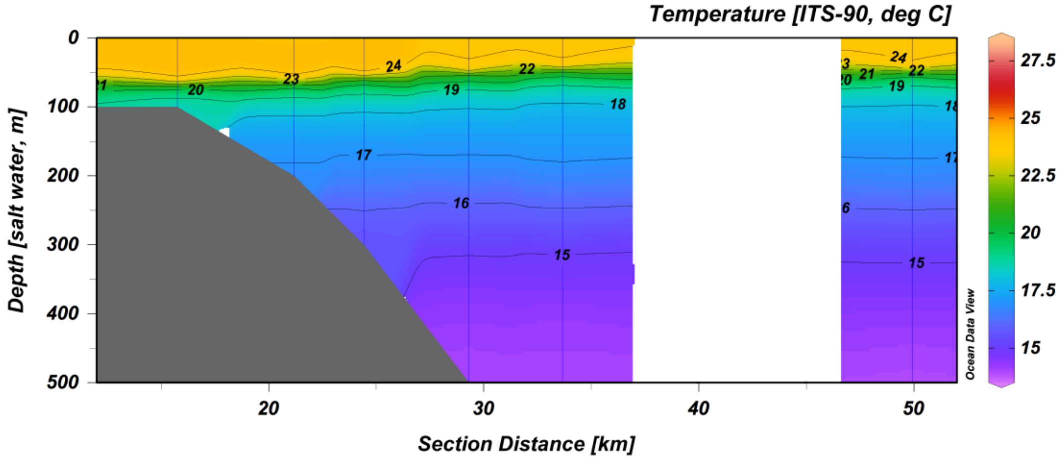

Spring 2016

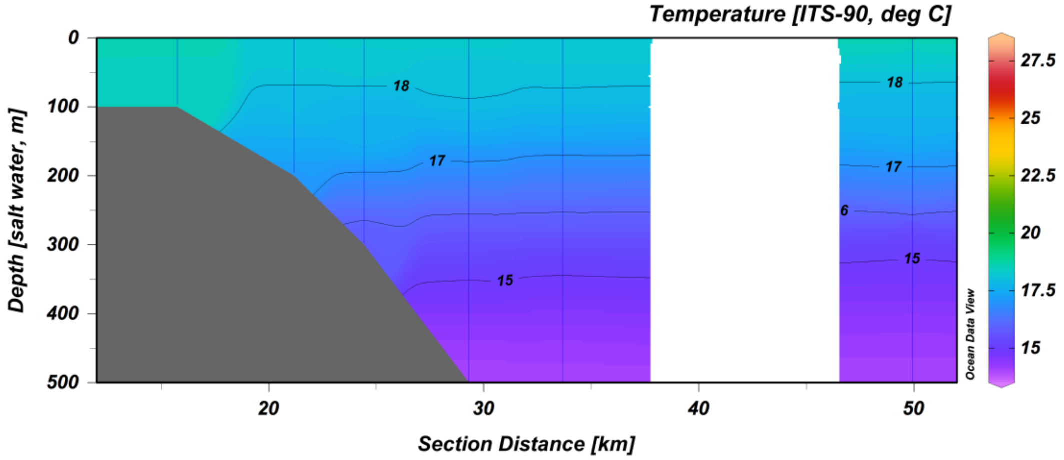

Summer 2016

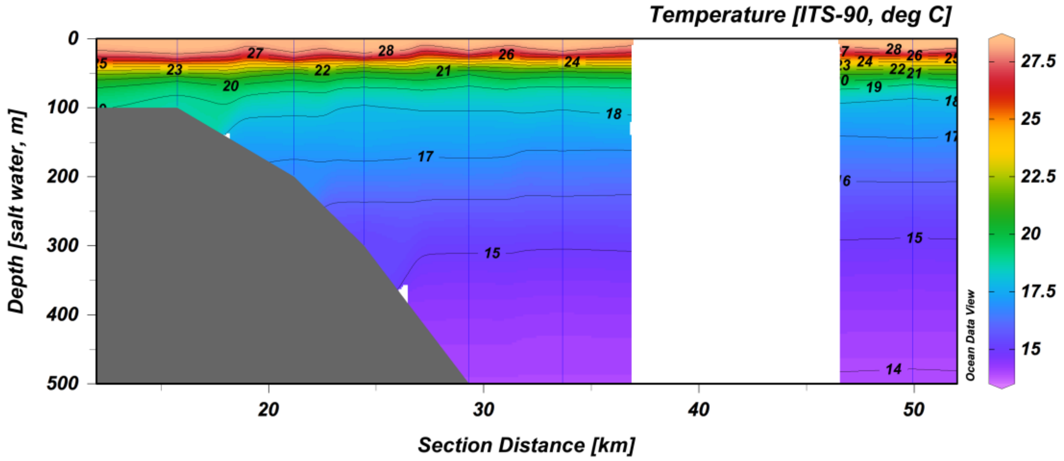

Supplementary Figure S1 B: Salinity - depth profiles of based on CTD data. Due to technical problems, no salinity data were available for station 1 in the spring 2016 cruise. For software settings see A. Contours indicate increments of 0.05 PSU. Color scale is the same for all plots.

Winter 2014

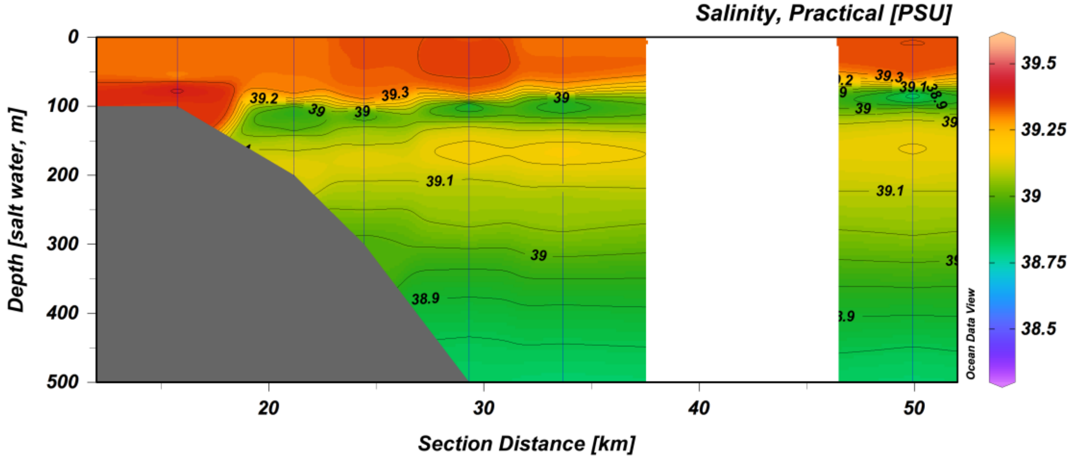

Spring 2015

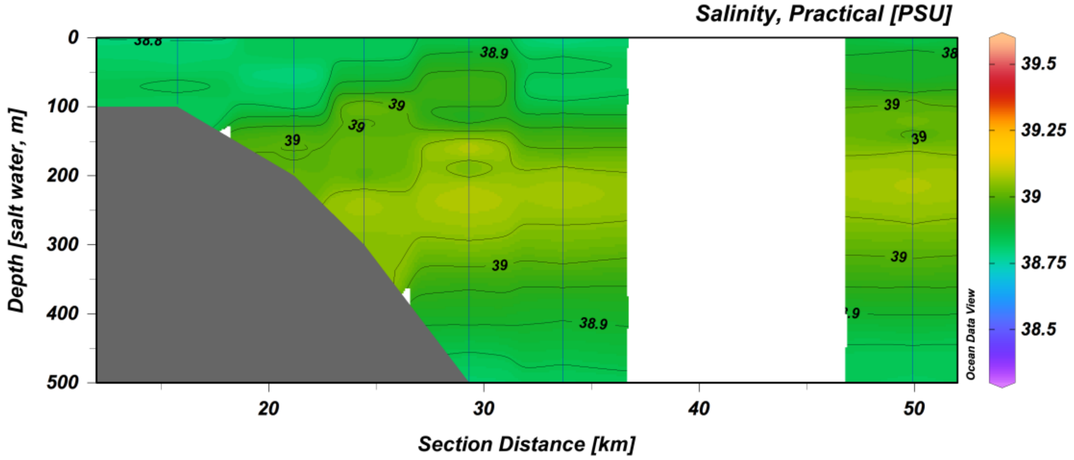

Summer 2015

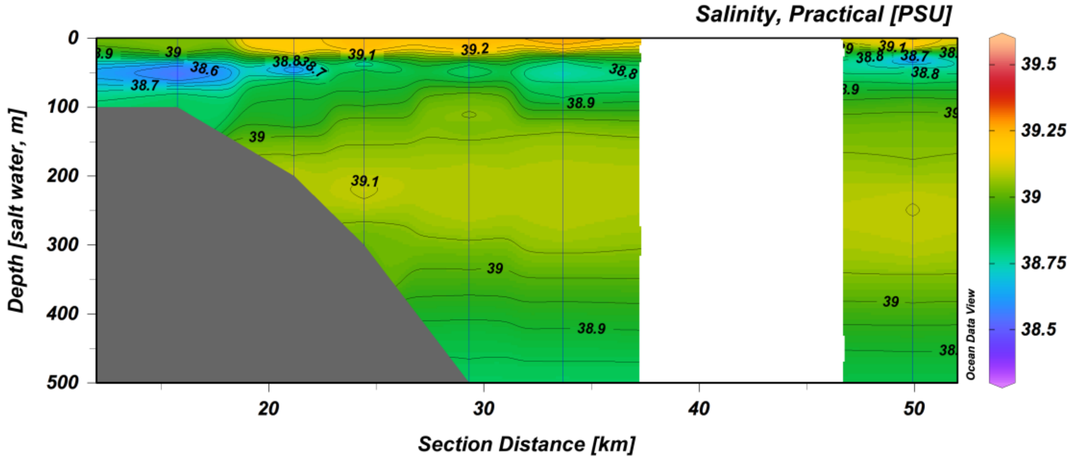

Supplementary Figure S1 B: continued.

Winter 2015

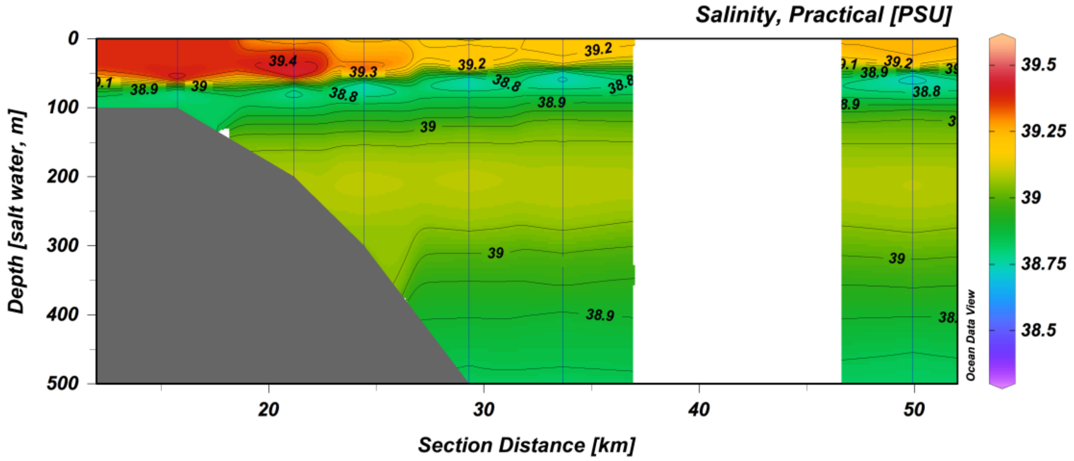

Spring 2016

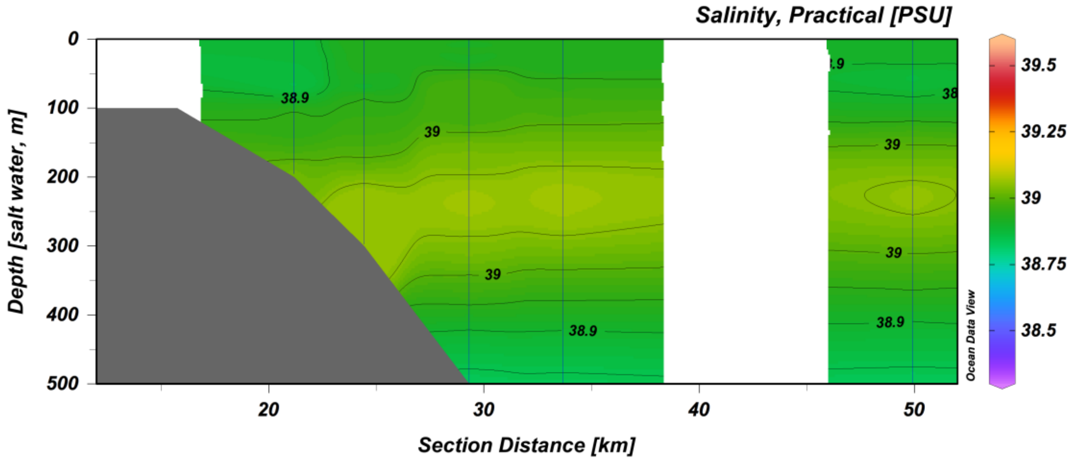

Summer 2016

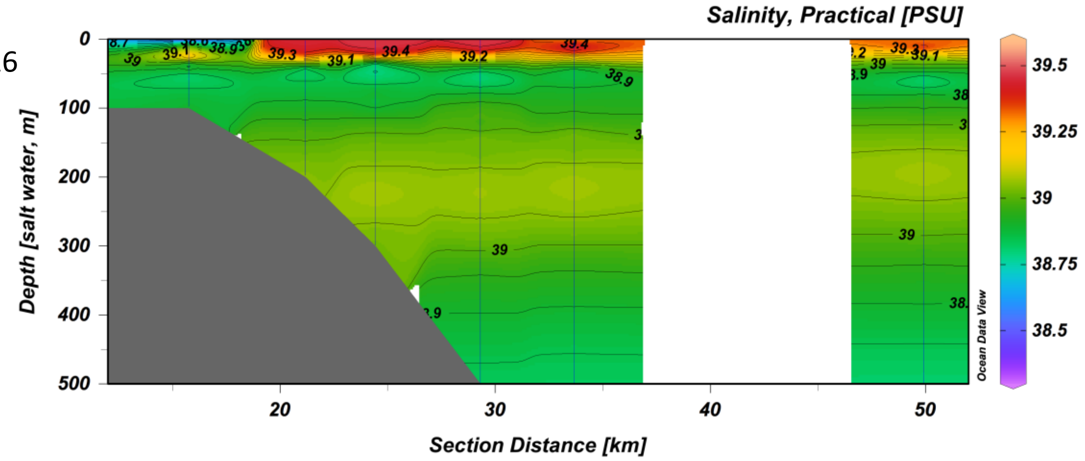

Supplementary Figure S1 C: Chlorophyll *a* fluorescence – depth profiles based on CTD data. For software settings see A. Contours indicate increments of 0.05. Color scale is the same for all plots.

Winter 2014

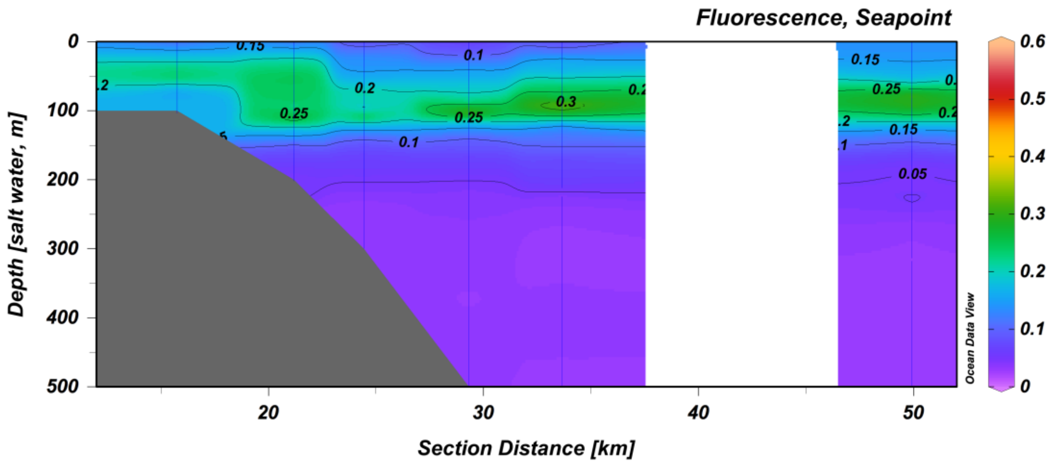

Spring 2015

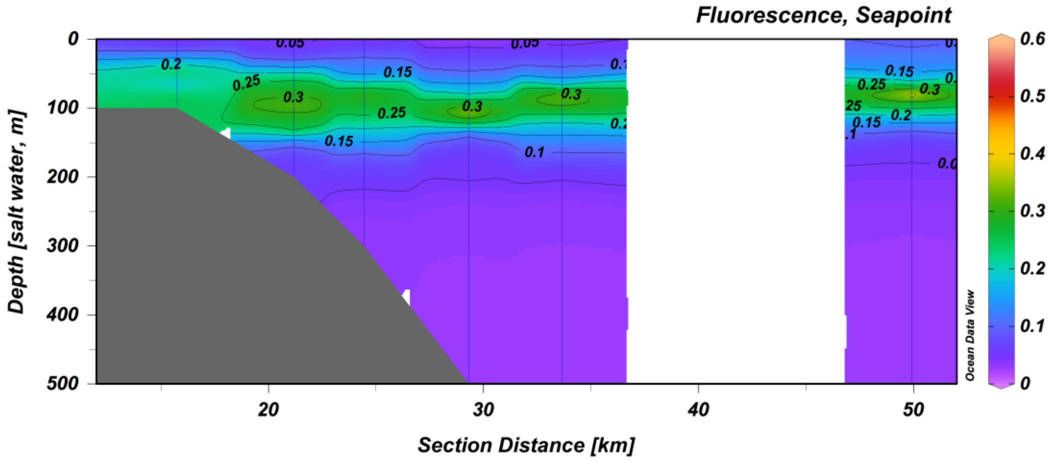

Summer 2015

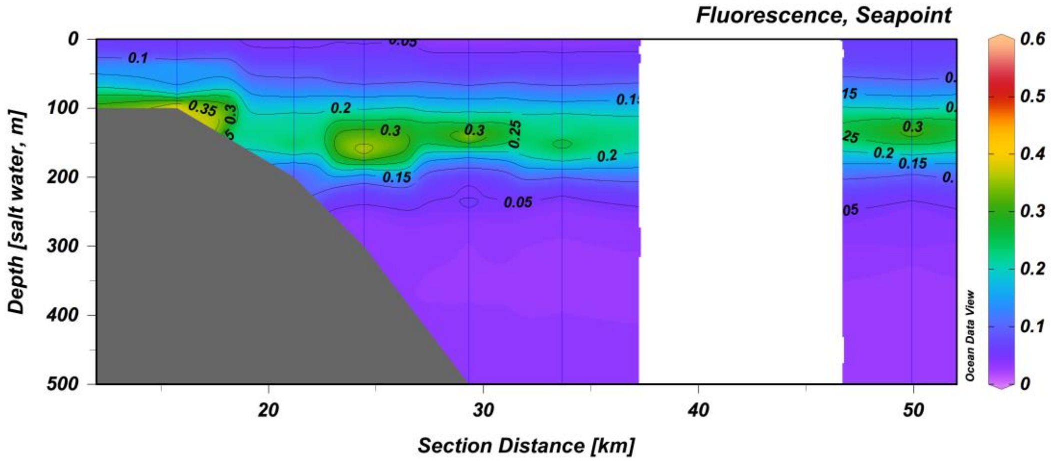

Supplementary Figure S1 C: continued.

Winter 2015

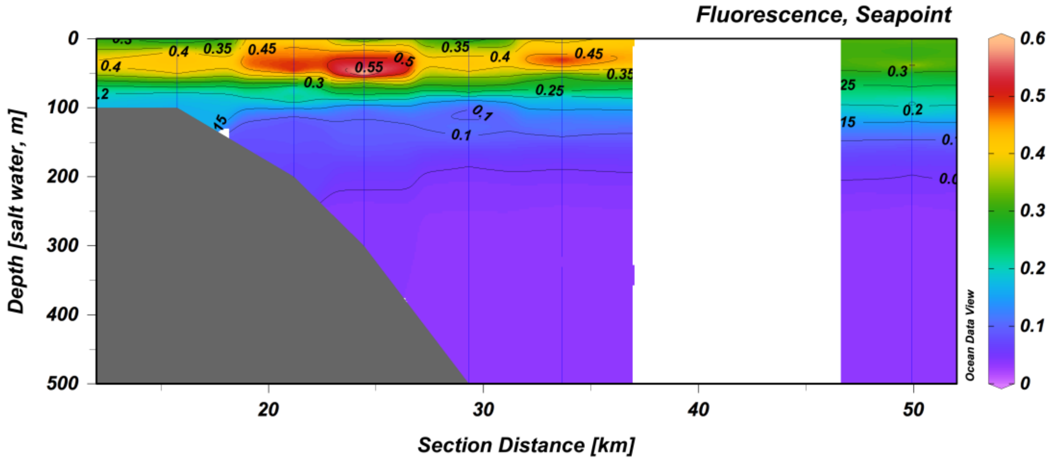

Spring 2016

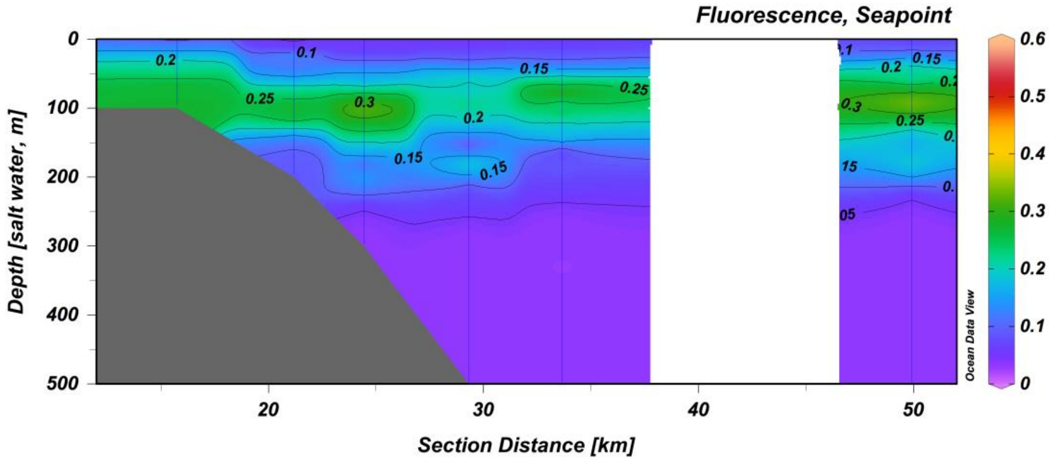

Summer 2016

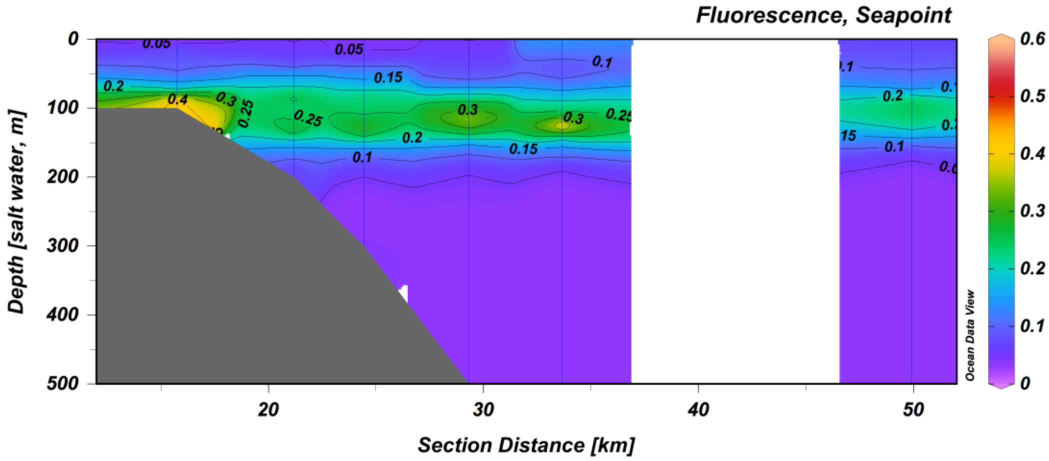

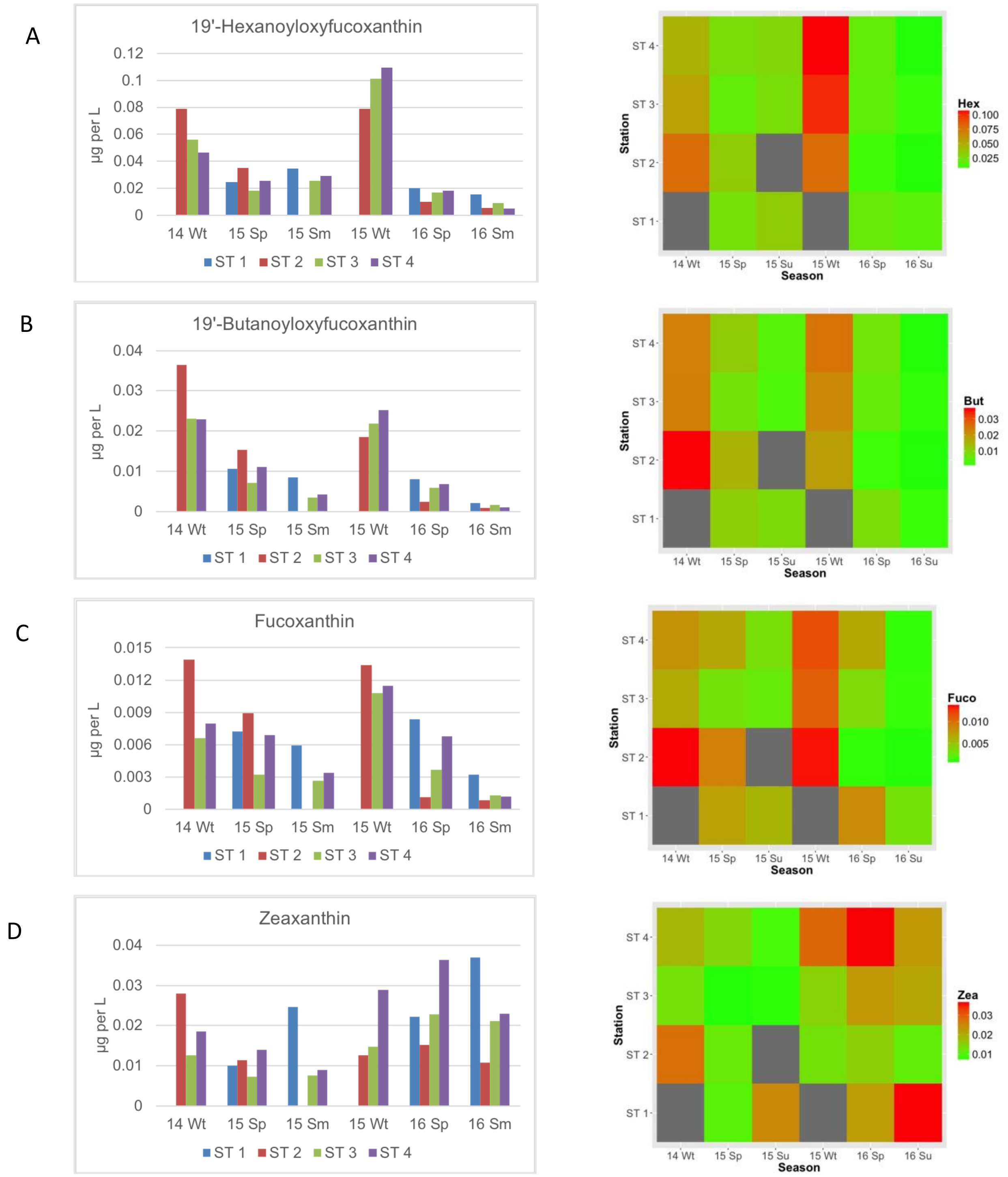

Supplementary Figure S2: Concentrations of the main detected diagnostic pigments: A) 19'-hexanoyloxyfucoxanthin, B) 19'-butanoyloxyfucoxanthin, C) fucoxanthin, D) zeaxanthin. No pigment samples were available for station 1 in early the winter 2014 and 2015 and for station 2 in the summer 2015 as indicated by the grey tiles in the heat maps.

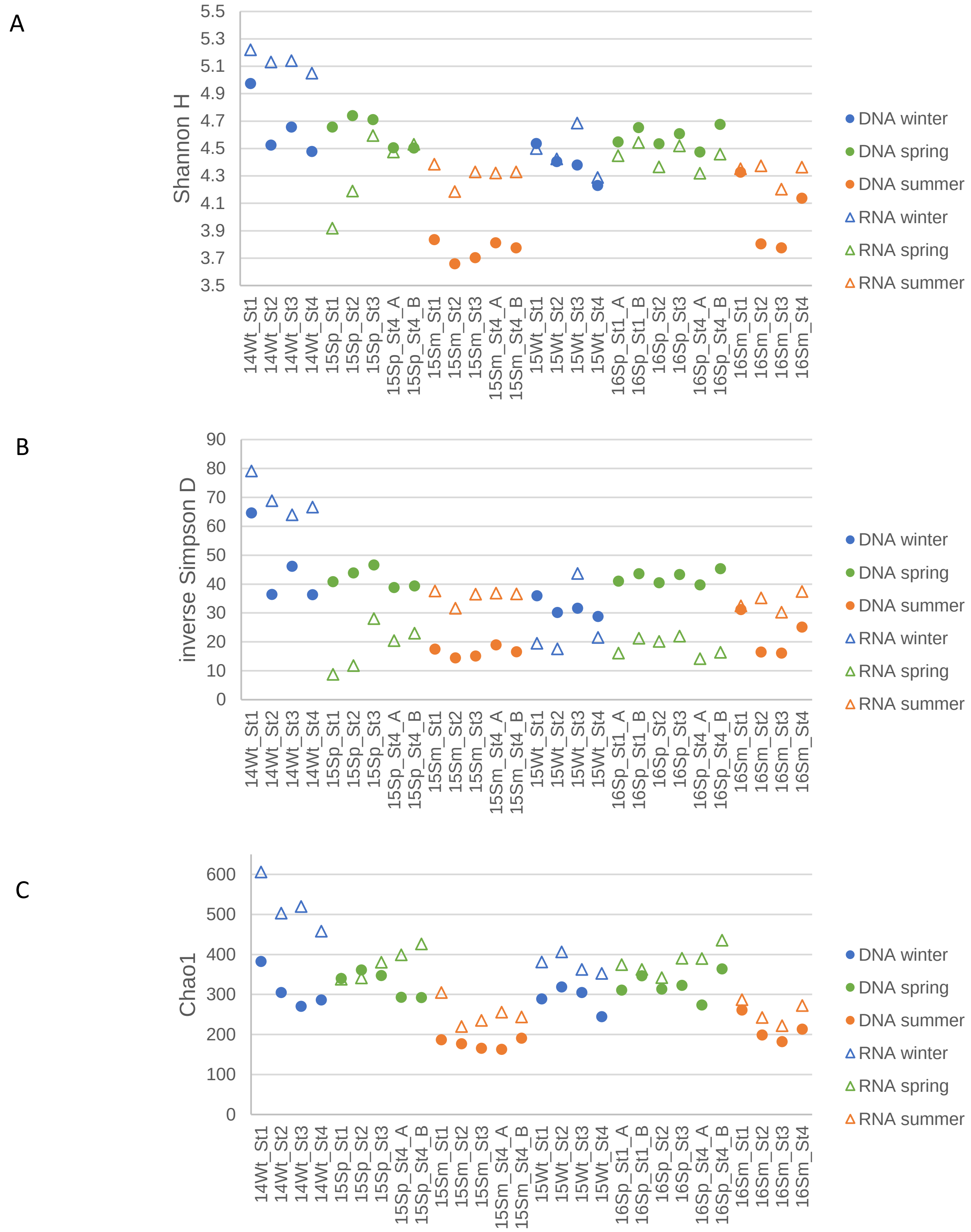

Supplementary Figure S3: Seasonal difference in diversity indices. A) Shannon, B) inverse Simpson, C) Chao1. Stations are sorted from station 1 to 4 (left to right) along the x-axis within each cruise. For statistical tests only the A replicates were used. Significant seasonal differences

were found in all three alpha diversity indices for both resident and active communities (Kruskal-Wallis test, $P<0.01$ in all six seasonal comparisons). DNA samples: In all three indices summer samples were significantly lower than spring and early winter samples (Dunn's post hoc test, Bonferroni corrected $P<0.05$), with no difference between the latter two.

In RNA samples: Shannon diversity was significantly lower in summer samples than early winter samples (Dunn's post hoc test, Bonferroni corrected $P<0.05$) with spring samples not differing from summer nor early winter samples. Chao1 indices of summer sample were significantly lower than those of spring and early winter samples (Dunn's post hoc test, Bonferroni corrected $P<0.05$) with no difference between early winter and spring samples; inverse Simpson diversity was significantly lower in spring samples compared to summer and early winter samples (Dunn's post hoc test, Bonferroni corrected $P<0.05$) with no differences between the latter two.

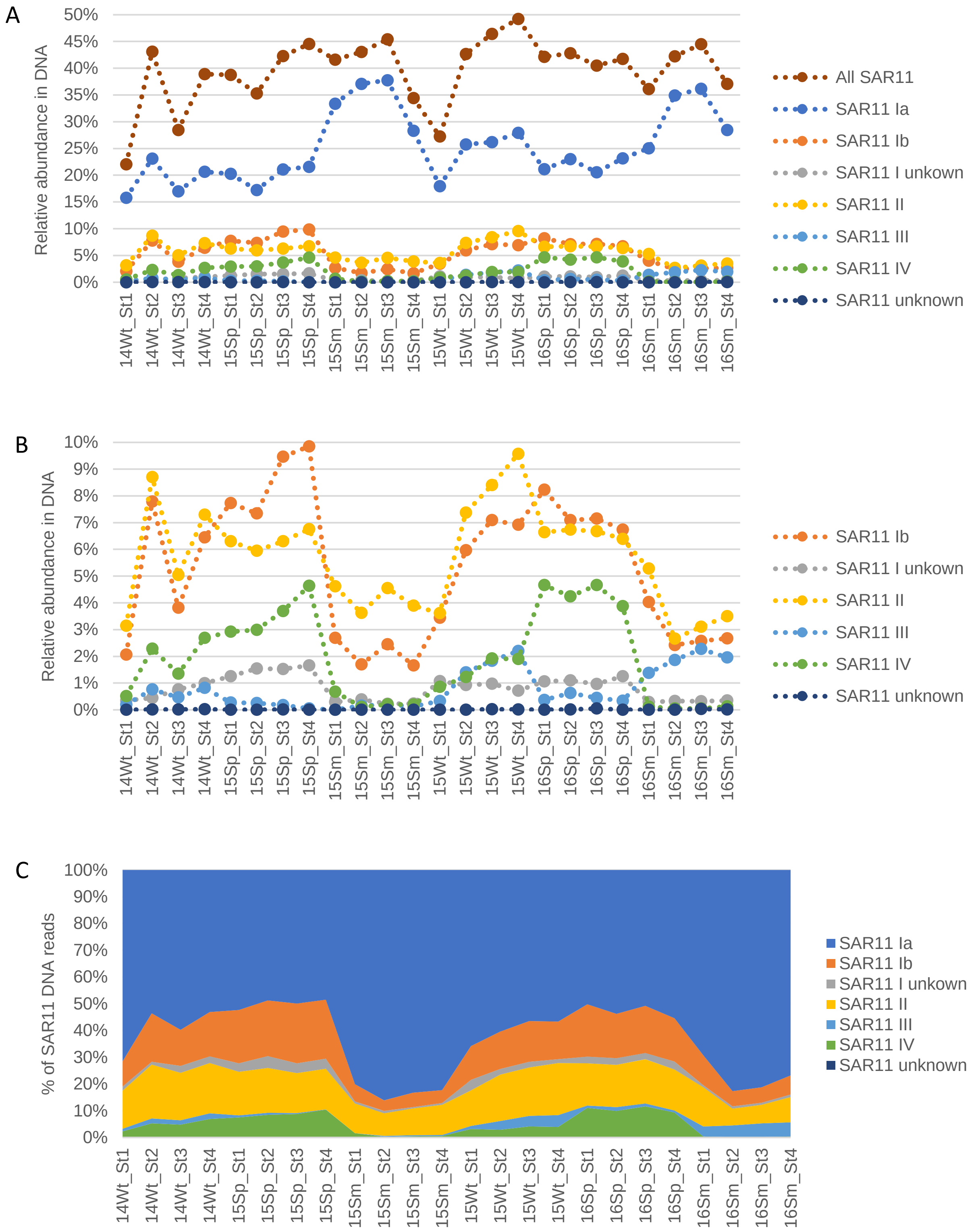

Supplementary Figure S4: SAR11 relative abundance based on DNA data. A) SAR11 order and clade I subclades; B) non-clade Ia SAR11 clades. C) Contribution of different clades to all SAR11 DNA reads. Sample code: Last two digit of sampling year, season (Wt: winter, Sp: spring, Sm: summer), station (St1 most coastal, St4 most offshore).

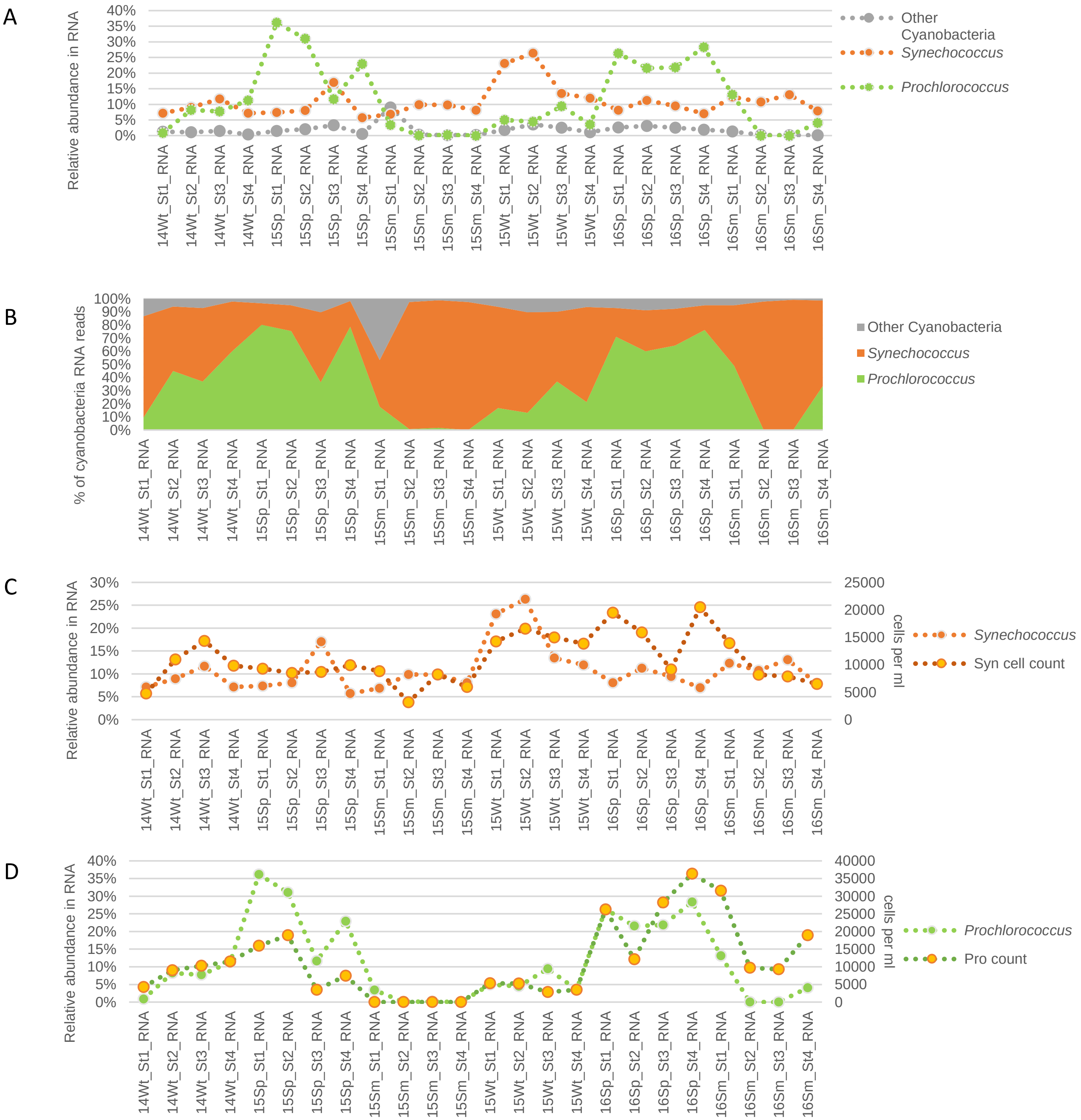

Supplementary Figure S5: Cyanobacteria activity and abundance. **A**) Cyanobacteria abundance in RNA samples and **B**) composition of cyanobacteria RNA reads. Note that a single *Trichodesmium* ESV is responsible for the rise in other cyanobacteria in the station 1 in summer 2015. It was not detected in the DNA samples that are 5 µm pre-filtered. Comparisons of **C**) *Synechococcus* and **D**) *Prochlorococccus* based on relative abundance of RNA reads and cell

counts from flow cytometry. Sample code: Last two digit of sampling year, season (Wt: winter, Sp: spring, Sm: summer), station (St1 most coastal, St4 most offshore).

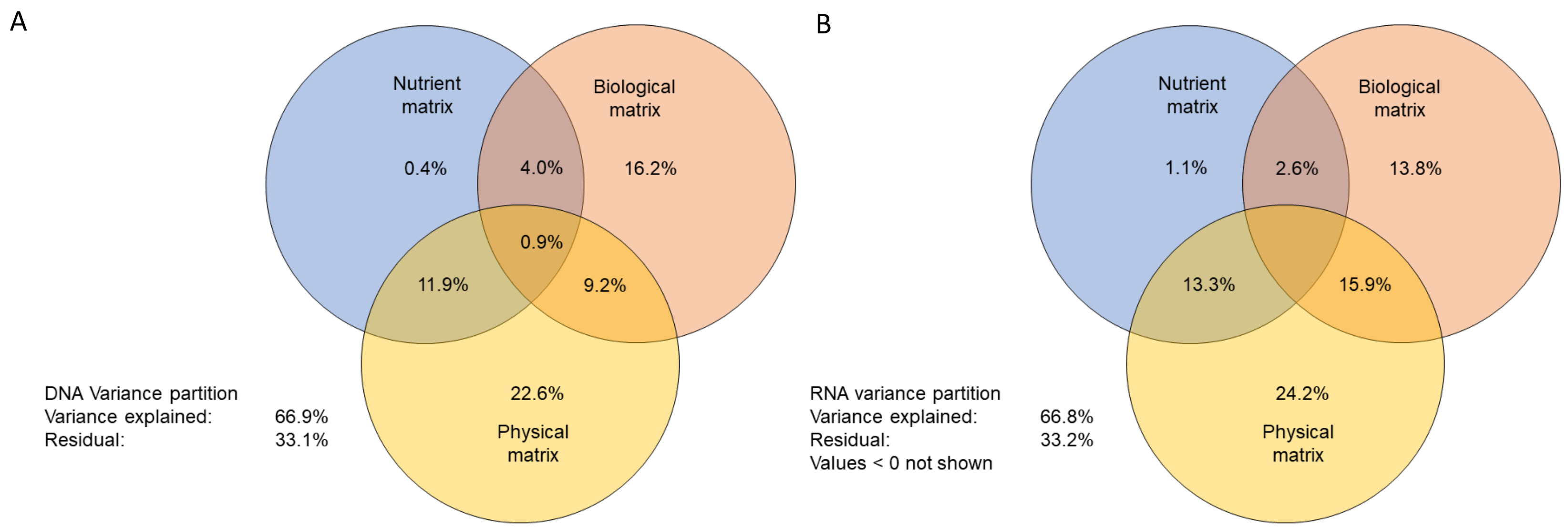

Analysis		DNA			RNA		
		Physical	Biological	Nutrients	Physical	Biological	Nutrients
Variation partitioning	Total VE (%)	44..5	30.2	17.1	49.4	28.3	13.0
	Independent VE (%)	22.6	16.2	4.2	24.2	13.8	1.1
dbRDA	Inertia explained (%)	54.2***	39.3***	27.9**	58.2***	37.6***	24.3**
	Significant variables	Temperature***<br>Salinity**	Picoeukaryotes***<br>Fluorescence***<br>Total cell count**	Phosphate*<br>NO3+NO2*	Temperature***<br>Salinity**<br>Turbidity*	Picoeukaryotes***<br>Fluorescence***<br>Total cell count**	Phosphate*<br>NO3+NO2*
conditioned dbRDA	Inertia explained (%)	22.8***	15.8***	4.8 ns	23.6***	13.9**	5.1 ns
	Significant variables	Temperature***<br>Salinity*	Fluorescence***<br>Total cell count*	ns	Temperature ***<br>Salinity**	Fluorescence***<br>Total cell count*	ns

Supplementary Figure S6: Variation in microbial community composition explained by the physical, biological, and nutrient matrixes. **A**) DNA and **B**) RNA Venn diagrams of the explained variation by matrix based on variation partitioning analyses on the Bray-Curtis community matrixes. The table shows the significance and the significant factors of each matrix in the distance-based redundancy analyses (dbRDA) analyses and the

conditioned dbRDA, which was conditioned by the factors of the other two matrices. dbRDA was run following McArdle and Anderson 2001 as implemented in the R package vegan. Asterisks indicate significance level for the model and factors: * $p<0.05$; ** $p<0.01$; ***$p<0.001$; ns not significant.

Supplementary Table S1: Station locations, bottom depth, distance to shore and distances between stations. Station 1A and 2B were sampled to identify gradients in the CTD data and from the second cruise onwards also in the nutrients.

Station	Location (NMEA)	Bottom depth (m)	Distance (km) to					
			Herzliya Marina	ST 1	ST 1A	ST 2	ST 2B	ST 3
ST 1	32 15.24 N<br>034 39.53 E	100	16.13	*				
ST 1A	32 17.07 N<br>034 36.89 E	200	21.48	5.35	*			
ST 2	32 18.26 N<br>034 35.07 E	300	25.08	8.95	3.60	*		
ST 2B	32 20.09 N<br>034 32.66 E	600	30.15	14.02	8.67	5.07	*	
ST 3	32 21.32 N<br>034 30.62 E	800	34.06	17.93	12.58	8.98	3.91	*
ST 4	32 26.99 N<br>034 22.51 E	1200	50.54	34.41	29.06	25.46	20.39	16.48

Supplementary Table S2: Nutrient data in µmol/l of samples collected at 10 m. Detection limits are 0.05 µmol/l for silica and nitrate+nitrite and 0.04 µmol/l for phosphate. Nutrients were measured in duplicates. From Spring 2015 onwards nutrients were also measured at the intermediate stations 1A and 2B to improve detection of potential gradients.

Year Season	Station	Phosphate		Nitrate+Nitrite		Silicate	
		Average	SD	Average	SD	Average	SD
2014 Winter	ST 1	0.07	0.01	0.69	0.37	2.10	0.05
	ST 2	0.01	0.01	0.34	0.14	0.47	0.15
	ST 3	0.03	0.03	0.32	0.19	0.50	0.27
	ST 4	0.06	0.00	0.18	0.07	0.63	0.06
2015 Spring	ST 1	0.05	0.00	0.19	0.02	1.02	0.06
	ST 1A	0.04	0.00	0.26	0.04	0.84	0.00
	ST 2	0.07	0.01	0.34	0.06	0.52	0.01
	ST 2B	0.04	0.00	0.48	0.03	0.61	0.08
	ST 3	0.04	0.00	0.64	0.03	0.40	0.01
	ST 4	0.03	0.00	0.10	0.00	0.66	0.01
2015 Summer	ST 1	0.05	0.01	0.12	0.04	0.54	0.00
	ST 1A	0.04	0.01	0.04	0.00	0.51	0.00
	ST 2	0.04	0.01	0.05	0.00	0.52	0.00
	ST 2B	0.04	0.00	0.02	0.00	0.54	0.03
	ST 3	0.03	0.00	0.04	0.00	0.58	0.05
	ST 4	0.04	0.00	0.04	0.01	0.56	0.01
2015 Winter	ST 1	0.04	0.01	0.30	0.01	0.86	0.03
	ST 1A	0.05	0.00	0.16	0.00	0.77	0.00
	ST 2	0.04	0.00	0.28	0.12	0.66	0.00
	ST 2B	0.03	0.00	0.19	0.04	0.55	0.03
	ST 3	0.02	0.00	0.11	0.01	0.62	0.02
	ST 4	0.02	0.00	0.23	0.02	0.60	0.00
2016 Spring	ST 1	0.04	0.00	0.05	0.01	0.86	0.01
	ST 1A	0.04	0.00	0.04	0.01	0.60	0.02
	ST 2	0.04	0.01	0.04	0.00	0.59	0.07
	ST 2B	0.03	0.00	0.06	0.01	0.68	0.01
	ST 3	0.03	0.00	0.48	0.04	0.83	0.04
	ST 4	0.03	0.01	0.22	0.02	0.87	0.00
2016 Summer	ST 1	0.03	0.00	0.03	0.01	1.00	0.11
	ST 1A	0.02	0.01	0.02	0.01	0.64	0.02
	ST 2	0.01	0.01	0.04	0.00	0.64	0.04
	ST 2B	0.02	0.00	0.03	0.00	0.68	0.01
	ST 3	0.01	0.01	0.03	0.00	0.67	0.03
	ST 4	0.02	0.01	0.04	0.01	0.71	0.02

Supplementary Table S3: Nutrient data (phosphate, ammonium and nitrate+nitrite) of the July 2016 cruise from fresh filtered samples analyzed with 24h of collection. Silicate was measured on filtered acidified samples which were unfrozen 5 days before analysis. All ammonium and phosphate concentrations are below effective detection limits.

Cruise	Station	Phosphate (nM)	Nitrate (nM)	Ammonia (nM)	Silicate (µM)
Summer 2016 (fresh samples)	ST 1	5.1	43.7	4.2	1.52
	ST 1A	0.2	20	1.5	0.92
	ST 2	0.7	15.5	1.3	0.85
	ST 2B	2.1	28.8	6.1	0.8
	ST 3	1.4	17.3	5	0.83
	ST 4	1.4	8.4	7	0.75

Supplementary Table S4: Pigment concentrations in ng/l based on UPLC measurements.

Abbreviations for pigments: Chl *a*: chlorophyll *a*, 19-Hx: 19'-hexanoyloxyfucoxanthin, 19-Bx: 19'-butanoyloxyfucoxanthin, Fuco: fucoxanthin, Per: peridinin, DV-Chl *a*: divinyl chlorophyll *a*, Chl C2a: chlorophyll *c* peak 1, Chl C2b: chlorophyll *c* peak 2 (Chl C2 was separated as two peaks due to running conditions), Chl *b*: chlorophyll *b*, b-Car: beta-carotene, Zea: zeaxanthin, Diadino: diadinoxanthin, Diato: diatoxanthin, Dino: dinoxanthin. Other abbreviations: - : no sample, ND: not detected

Year Season	Station	Chl *a*	19-Hx	19-Bx	Fuco	Per	DV-Chl *a*	Chl C2a	Chl C2b	Chl *b*	b-Car	Zea	Diadino	Diato	Dino
2014 Winter	ST 1	-	-	-	-	-	-	-	-	-	-	-	-	-	-
	ST 2	128.53	78.96	36.45	13.89	ND	ND	8.93	12.65	ND	ND	28.00	3.54	ND	0.75
	ST 3	44.04	55.94	23.04	6.57	ND	ND	15.49	ND	ND	2.98	12.67	1.70	ND	ND
	ST 4	39.94	46.43	22.98	7.98	ND	ND	4.89	4.36	ND	ND	18.55	1.98	ND	0.44
2015 Spring	ST 1	57.31	24.52	10.63	7.22	ND	ND	4.28	4.43	ND	ND	9.97	2.69	ND	0.66
	ST 2	47.79	35.19	15.33	8.94	ND	0.44	9.13	10.33	0.86	ND	11.49	3.31	0.57	0.93
	ST 3	47.80	18.16	7.18	3.19	ND	ND	4.70	8.66	ND	ND	7.22	2.44	0.41	0.67
	ST 4	58.37	25.76	10.07	6.86	2.78	ND	4.87	7.99	ND	ND	13.98	2.72	ND	0.97
2015 Summer	ST 1	88.59	34.81	8.43	5.93	ND	ND	4.39	7.81	ND	ND	24.68	3.40	0.63	0.87
	ST 2	-	-	-	-	-	-	-	-	-	-	-	-	-	-
	ST 3	37.40	25.36	3.53	2.65	1.56	ND	2.24	2.22	ND	ND	7.59	2.10	1.14	0.84
	ST 4	58.53	29.28	4.17	3.36	2.94	ND	1.92	2.36	1.20	ND	8.99	2.94	1.77	1.12

Year Season	Station	Chl *a*	19-Hx	19-Bx	Fuco	Per	DV-Chl *a*	Chl C2a	Chl C2b	Chl *b*	b-Car	Zea	Diadino	Diato	Dino
2015 Winter	ST 1	-	-	-	-	-	-	-	-	-	-	-	-	-	-
	ST 2	160.86	78.67	18.57	13.39	ND	ND	10.00	9.27	ND	ND	12.58	5.72	ND	ND
	ST 3	153.61	101.34	21.85	10.80	ND	ND	11.83	11.92	ND	ND	14.67	7.52	ND	3.57
	ST 4	473.63	109.38	25.16	11.48	ND	ND	12.95	13.75	ND	2.08	28.85	13.28	ND	7.72
2016 Spring	ST 1	127.53	19.95	8.09	8.36	5.08	1.86	2.40	3.18	1.38	ND	22.26	ND	1.18	ND
	ST 2	84.22	9.95	2.38	1.12	ND	2.04	ND	0.80	0.94	0.60	15.27	2.92	1.65	ND
	ST 3	77.29	16.81	5.84	3.65	2.27	1.26	ND	0.96	3.73	0.33	22.81	4.49	1.53	ND
	ST 4	179.22	18.42	6.86	6.76	2.81	9.06	ND	2.47	2.71	0.69	36.29	4.78	1.79	0.65
2016 Summer	ST 1	156.92	15.41	2.10	3.19	0.75	0.54	0.81	2.32	0.99	ND	37.00	7.81	0.64	ND
	ST 2	55.47	5.58	0.94	0.86	0.20	ND	0.28	0.67	ND	ND	10.78	3.99	0.56	ND
	ST 3	129.44	8.93	1.57	1.27	ND	ND	0.56	1.46	1.70	0.92	21.08	6.16	1.02	ND
	ST 4	177.49	5.20	0.98	1.20	1.67	ND	0.66	1.47	4.43	ND	22.90	6.52	0.80	2.21

Supplementary Table S5: CTD data at 10m for the stations sampled for DNA and RNA. Due to technical problems, no salinity value was obtained for station 1 in the Spring 2016 cruise. For the environmental matrix, the value from station 1A was used and is given in brackets.

Year Season	Station	Temperature [°C]	Salinity [PSU]	Fluorescence	Turbidity [FTU]
2014 early Winter	ST 1	22.57	39.32	0.140	0.151
	ST 2	22.43	39.34	0.108	0.149
	ST 3	22.47	39.31	0.096	0.131
	ST 4	22.43	39.35	0.117	0.131
2015 Spring	ST 1	17.86	38.82	0.068	0.161
	ST 2	17.73	38.83	0.061	0.117
	ST 3	17.64	38.81	0.053	0.066
	ST 4	17.72	38.87	0.083	0.085
2015 Summer	ST 1	26.22	39.04	0.063	0.832
	ST 2	26.68	39.24	0.052	1.341
	ST 3	26.52	39.27	0.042	1.022
	ST 4	26.59	39.24	0.045	1.390
2015 early Winter	ST 1	24.37	39.38	0.509	0.208
	ST 2	24.27	39.26	0.436	0.094
	ST 3	24.04	39.21	0.400	0.090
	ST 4	24.08	39.24	0.277	0.073
2016 Spring	ST 1	18.59	(38.88)	0.136	0.206
	ST 2	18.24	38.93	0.067	0.143
	ST 3	18.22	38.93	0.068	0.117
	ST 4	18.44	38.91	0.092	0.215
2016 Summer	ST 1	28.43	39.17	0.076	6.219
	ST 2	28.40	39.47	0.051	3.895
	ST 3	28.42	39.46	0.133	3.897
	ST 4	28.66	39.39	0.075	3.967

Supplementary Table S6: Flow cytometry data in cells/ml. Samples were all run on a BD cantor flow cytometer. Samples for total cell counts (heterotrophs + autotrophs) were stained with Sybr Green. Samples for *Prochlorococcus*, *Synechococcus* and Picoeurkaryotes were not stained and counted base on autofluorescence signals. Samples were run in triplicates and technical replicates were run for each sample. ND: not detected

Year Season	Station	*Prochloroccous*		*Synechococcus*		Picoeukaryotes		Total cells	
		Average	SD	Average	SD	Average	SD	Average	SD
2014 early Winter	ST 1	4223	102	4800	266	39091	4052	538408	85443
	ST 2	9022	464	10978	632	4106	2395	619542	80394
	ST 3	10303	1136	14330	792	6109	765	634490	112194
	ST 4	11486	1188	9824	571	5445	2836	939931	435038
2015 Spring	ST 1	15966	1777	9302	1116	ND		897962	420694
	ST 2	18913	410	8534	880	ND		1008005	218981
	ST 3	3498	554	8717	328	ND		977237	162732
	ST 4	7477	1137	9904	1585	ND		481180	34865
2015 Summer	ST 1	ND		8818	2666	ND		259249	23515
	ST 2	ND		3187	131	ND		207478	10469
	ST 3	ND		8259	1838	ND		234948	38054
	ST 4	ND		5983	889	13939	7981	176812	19239
2015 early Winter	ST 1	5319	662	14266	346	4021	211	459085	144217
	ST 2	5262	737	16589	6296	5496	269	417091	21952
	ST 3	2851	1026	14979	6569	4000	1366	325249	52443
	ST 4	3468	334	13837	5596	4362	391	295332	138239
2016 Spring	ST 1	26182	1639	19494	1013	ND		466767	54832
	ST 2	12092	674	15880	681	ND		576033	26706
	ST 3	28175	23272	9165	1382	ND		506129	18949
	ST 4	36318	517	20475	1291	ND		562133	13642
2016 Summer	ST 1	31546	6119	13946	486	15348	6312	1043058	182054
	ST 2	9736	346	8194	235	1927	206	397337	8553
	ST 3	9271	416	7854	224	2723	957	419149	48082
	ST 4	18942	6726	6509	560	8120	3172	233578	18835

Supplementary Table S7: Relation between relative abundance in RNA and DNA samples for families contributing >= 1% of total reads in either sample type. SAR11 clade III is below the 1% threshold but was added for comparison. Significant P-values are in bold.

Taxonomy	Average RNA:DNA ratio	Standard deviation	Spearman rho	*P*-value
Proteobacteria; SAR11 clade I	0.36	0.09	0.663332	**0.00041**
Proteobacteria; SAR11 clade II	0.16	0.05	0.647819	**0.00062**
Proteobacteria; SAR11 clade III	0.28	0.16	0.890095	**5.81E-09**
Proteobacteria; SAR11 clade IV	0.93	0.59	0.942232	**6.30E-12**
Proteobacteria; AEGEAN-169_marine_group	1.08	0.38	0.630743	**0.00095**
Proteobacteria; Rhodobacteraceae	1.56	0.82	0.224646	0.29130
Proteobacteria; S25-593	1.77	0.67	0.609174	**0.00158**
Proteobacteria; SAR116_clade	1.83	0.53	0.820899	**8.92E-07**
Proteobacteria; Alteromonadaceae	8.95	8.49	0.882945	**1.12E-08**
Proteobacteria; Ectothiorhodospiraceae	3.69	1.97	0.857541	**8.61E-08**
Proteobacteria; KI89A_clade	2.45	0.73	0.636059	**0.00084**
Proteobacteria; SAR86_clade	1.17	0.27	0.722682	**0.00007**
Actinobacteriota; Actinomarinaceae	0.02	0.02	0.023874	0.91180
Cyanobacteriiota; Cyanobiaceae	2.48	0.91	0.756745	**0.00002**
BacteroidotaFlavobacteriaceae	0.20	0.09	0.312323	0.13730
Marinimicrobiota; Marinimicrobia (SAR406)	0.76	0.45	0.945888	**3.12E-12**
Verrucomicrobiota; Arctic97B-4_marine_group	16.28	11.71	0.898178	**2.61E-09**
Verrucomicrobiota; Puniceicoccaceae	1.69	0.68	0.736246	**0.00004**

Supplementary Table S8: Statistics of the two permutational multivariate analyses of variance using Bray-Curtis distances and 999 permutations performed using the Adonis function of the R package vegan. Significant factors are highlighted in bold.

Sample type		Df	Sums of Sqaures	Mean Squares	F.Model	R^2	Pr(>f)
RNA	**Season**	2	2.0648	1.03240	13.0405	0.54948	**0.001**
	Station	3	0.3266	0.10887	1.3751	0.08692	0.177
	Season:Station	6	0.4163	0.06938	0.8763	0.11078	0.632
	Residual	12	0.9500	0.07917		0.25282	
	Total	23	3.7577			1.00000	
DNA	**Season**	2	1.31252	0.65626	13.1301	0.55944	**0.001**
	Station	3	0.17752	0.05917	1.1839	0.07566	0.310
	Season:Station	6	0.25631	0.04272	0.8547	0.10925	0.659
	Residual	12	0.59977	0.04998		0.25564	
	Total	23	2.34612			1.00000	

Supplementary Table S9: Comparison of SAR11 relative abundance of this study to literature data.

Study area	Location	Abundance %	Method	Reference
Southeastern Mediterranean Sea	Offshore	41 ± 5 (range 28 to 49)	Amplicon sequencing	this study
Sargasso Sea, North Atlantic gyre	Offshore	33 ± 8	FISH	Carlson et al. 2009
South Atlantic gyre	Offshore	Up to 36 ± 9	CARD-FISH	Morris et al. 2012
North Pacific gyre	Offshore	33 to 44	qPCR	Eiler et al. 2009
South Pacific gyre	Offshore	up to 53	Amplicon sequencing	West et al. 2016
Southern Adriatic Sea	Offshore	47-59*	CARD-FISH, pyrosequencing	Korlević et al. 2015
Southeastern Mediterranean Sea	Coastal	22 to 42 (average 35)	Amplicon sequencing	this study
Northwestern Mediterranean Sea	Coastal	Up to 27 ± 8	CARD-FISH	Alonso-Sáez *et al.* 2007
Northwestern Mediterranean Sea	Coastal	33.9	Amplicon sequencing	Auladell et al. 2021
Adriatic Sea	Coastal	6.98 to 31.7	Clone library	Tinta et al. 2015
Southern Mediterranean Sea	Coastal	23.9	Amplicon sequencing	Quéméneur et al. 2020

*based on numbers reported at 0 and 10 m.
